# Selection of Negative Charged Acidic Polar Additives to Regulate Electric Double Layer for Stable Zinc Ion Battery

**DOI:** 10.1007/s40820-024-01475-5

**Published:** 2024-08-14

**Authors:** Xing Fan, Lina Chen, Yongjing Wang, Xieyu Xu, Xingxing Jiao, Peng Zhou, Yangyang Liu, Zhongxiao Song, Jiang Zhou

**Affiliations:** 1 State Key Laboratory for Mechanical Behavior of Materials, Xi’an Jiaotong University, Xi’an, 710049 People’s Republic of China; 2 School of Materials Science and Engineering, Harbin Institute of Technology (Shenzhen), Shenzhen, 518055 People’s Republic of China; 3 Research Institute of Frontier Science, Southwest Jiaotong University, Chengdu, 610031 People’s Republic of China; 4 Hunan Provincial Key Defense Laboratory of High Temperature Wear-Resisting Materials and Preparation Technology, Hunan University of Science and Technology, Xiangtan, 411201 People’s Republic of China; 5 School of Instrument Science and Technology, Xi’an Jiaotong University, Xi’an, 710049 People’s Republic of China; 6 School of Materials Science and Engineering, Hunan Provincial Key Laboratory of Electronic Packaging and Advanced Functional Materials, Central South University, Changsha, 410083 People’s Republic of China

**Keywords:** Aqueous Zn-ion batteries, Zn metal anode, Negative charged acidic polar additives, Electric double-layer regulation

## Abstract

Zinc-ion batteries are promising for large-scale electrochemical energy storage systems, which still suffer from interfacial issues, e.g., hydrogen evolution side reaction (HER), self-corrosion, and uncontrollable dendritic Zn electrodeposition. Although the regulation of electric double layer (EDL) has been verified for interfacial issues, the principle to select the additive as the regulator is still misted. Here, several typical amino acids with different characteristics were examined to reveal the interfacial behaviors in regulated EDL on the Zn anode. Negative charged acidic polarity (NCAP) has been unveiled as the guideline for selecting additive to reconstruct EDL with an inner zincophilic H_2_O-poor layer and to replace H_2_O molecules of hydrated Zn^2+^ with NCAP glutamate. Taking the synergistic effects of EDL regulation, the uncontrollable interface is significantly stabilized from the suppressed HER and anti-self-corrosion with uniform electrodeposition. Consequently, by adding NCAP glutamate, a high average Coulombic efficiency of 99.83% of Zn metal is achieved in Zn|Cu asymmetrical cell for over 2000 cycles, and NH_4_V_4_O_10_|Zn full cell exhibits a high-capacity retention of 82.1% after 3000 cycles at 2 A g^−1^. Recapitulating, the NCAP principle posted here can quicken the design of trailblazing electrolyte additives for aqueous Zn-based electrochemical energy storage systems.

**Supplementary Information:**

The online version contains supplementary material available at 10.1007/s40820-024-01475-5.

## Highlights

- Negative charged acidic polarity (NCAP) has been unveiled as the guideline for selecting additives to regulate electric double layer (EDL) for Zn-ion batteries.
- NCAP glutamate has been verified to regulate EDL structure with synergetic effects, including preferential adsorption on Zn anode and reconstruction of hydrated Zn-ion clusters.
- Adding NCAP additives, Zn|Cu half-cell achieves a high Coulombic efficiency of 99.83% for 2000 cycles, and NH_4_V_4_O_10_|Zn full cell realizes a high-capacity retention of 82.1% for 3000 cycles.

**Supplementary Information:**

The online version contains supplementary material available at 10.1007/s40820-024-01475-5.

## Graphical abstract

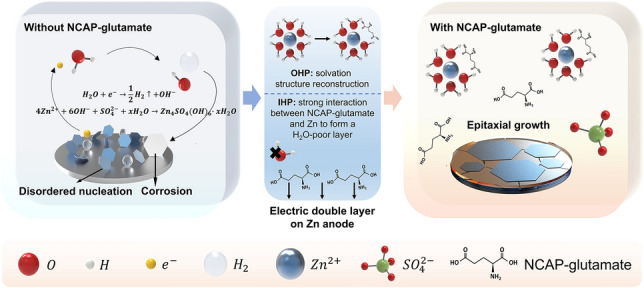

## Introduction

Renewable energy including solar, geothermal, wind, tidal energy, etc., is considered one of the most important for the energy crisis and environmental concerns [1, 2]. Whereas, its applications have been hampered by the intrinsically uneven spatio-temporal distribution and deployment [3]. Therefore, a large-scale electrochemical energy storage system is urgently needed to enable the intermittent power of renewable energy to become dispatchable. Metallic Zinc (Zn) embraces a high theoretical capacity of 820 mAh g^−1^ and a low reduction potential of − 0.76 V versus the standard hydrogen electrode [4], making aqueous Zinc-ion batteries (ZIBs) the most promising candidates for large-scale energy storage with superior safety performance, low economical cost, and environmental friendliness [5–7]. Nevertheless, ZIBs’ networking in smart grids with renewable energy is still challenged by the insufficient lifespan caused by the interfacial degradation of the Zn anode.

Indeed, the surface of the Zn anode encounters problematic issues like the geometric fluctuation caused by the growth of dendritic Zn [8–10], increased interfacial resistance from side reaction of hydrogen evolution reaction (HER) with the accumulation of passivating by-products of Zn_4_(SO_4_)_4_(OH)_6_⋅*x*H_2_O [11, 12], as well as the self-discharging from the spontaneous corrosion on Zn metal in acidic electrolyte [13]. Worse still, these issues could intensify each other. Essentially, these issues happen within the electric double layer (EDL) formed between the Zn metal anode and the aqueous electrolyte [14, 15], which consists of the inner Helmholtz plane (IHP) and outer Helmholtz plane (OHP) [16]. The IHP is composed of dissolved Zn-ions and free H_2_O molecules, and hydrogen gas is formed due to the combination of H_2_O and electrons on Zn anode, which is HER. The OHP consists of solvated hydrated, which provides the free H_2_O molecules in IHP. Thus, reducing H_2_O molecules in OHP and regulating the deposition of Zn-ions in IHP are necessary. In other words, the regulation of EDL structure plays a significant role in governing the Zn plating process and HER side reaction for improving the electrochemical performance of ZIBs, including anode modification [17–19], and electrolyte optimization [20–22]. Typically, electrolyte additives to regulate the hydrophobic zincophilic EDL structure should embrace properties such as lower adsorption energy on the Zn surface, higher affinity with Zn-ion as well as a stronger bond with H_2_O, thus changing the adsorption condition on Zn anode and the hydrated Zn^2+^ solvation structure in EDL to ensure uniform Zn deposition and suppressing HER side reaction with less passivation [23]. Very recently, massive additives sprung up to regulate the EDL structure selected from the perspective of polarity, like maltose [24], ethyl acetate [25], D-Arabinose [26], ectoine [27], sodium gluconate [28], ammonium hydroxide [28], etc. [29, 30]. Although advancements have been obtained to some extent, the discovery and selection of additives are somewhat accidental based solely on polarity, lacking a more detailed general principle to guide the efficient selection of additives as EDL regulators. Therefore, it is necessary to study the regulating mechanism of additives with the ability of solvation structure reconstruction and adsorption on the Zn surface, which can be regarded as an indispensable supplement for the guideline for selecting additives to reinforce the interfacial stability of the Zn metal anode.

Here, several typical H_2_O-soluble amino acids with different characteristics (Tab. S1**)** including negative-charged acidic polar glutamate (NCAP-Glu), positive-charged alkaline polar histidine (PCAP-His), non-polar glycine (NP-Gly), and non-ionic polar serine (NIP-Ser) were selected to explore the influence of dominating effect of solvation structure reconstruction or adsorption on Zn surface on the regulation of EDL [31]. Based on the theoretical and experimental comparison, an inner zincophilic H_2_O-poor layer is built on the Zn metal anode, and the replacement of H_2_O molecules of hydrated Zn^2+^ with additive is reconstructed using the selection NCAP-Glu. Consequently, the Zn|Cu asymmetrical cell can cycle with high Coulombic efficiency of 99.83% for over 2000 cycles, which enables NH_4_V_4_O_10_|Zn full cell to maintain a capacity as high as 82.1% of the initial capacity after 3000 cycles at 2 A g^−1^.

## Experimental

### Preparation of Electrolytes

ZnSO_4_·7H_2_O powders (Macklin, 99.995%) were dissolved in deionized (DI) water to obtain 2 M ZnSO_4_ solution as the control group. The chosen amino acids are: L-glutamate (C_5_H_9_NO_4_, Aladdin, 99.5%); L-histidine (C_6_H_9_N_3_O_2_, Aladdin, 99%); glycine (NH_2_CH_2_COOH, Aladdin, 98.5%); L-serien (HOCH_2_CH(NH_2_)CO_2_H, Aladdin, 99%).

### Preparation of Electrodes

Zn foil (thickness: 50 μm, 99.99%) was cut into a disc (*φ* = 12 mm) as the Zn anode electrode and the Cu foil (thickness: 50 μm, 99.99%) was cut into a disc (*φ* = 19 mm) as Cu cathode electrode to assemble asymmetrical cells. NH_4_V_4_O_10_ (NVO) powders used in full cells was synthesized by hydrothermal synthesis. In detail, 0.85 g ammonium vanadate (NH_4_VO_3_, Aladdin, 99%) was dissolved into DI water and stirred in a water bath at 80 °C for 40 min. After that, the color of the solution changed from white to pale yellow. Next, 0.85 g oxalic acid (H_2_C_2_O_4_·2H_2_O, Aladdin, 99%) powder was added into the solution and then continually stirred in the water bath at 80 °C for 30 min, and the color of the solution changed from white to pale blue-green. Poured the solution into a 50 mL Teflon-lined autoclave and heated at 180 °C for 8 h. After cooling, the reaction products were washed repeatedly with DI water and then dried at room temperature overnight to obtain the NVO powders. The cathode was prepared by mixing NVO powder, Ketjen black (KB), and polytetrafluorethylene (PTFE, 60 wt% dispersion in H_2_O, Canrd) with a mass ratio of 75:15:10 by isopropanol. Finally, the mixture was cast on 316 stainless steel wire and dried overnight at 60 °C.

### Characterizations

Raman spectra were characterized by the Laser Raman Spectrometer HORIBA. Different electrolytes were tested from 400 to 4000 cm^−1^. Nuclear magnetic resonance (NMR) was characterized by the equipment AVANCE III HD 600 MHz. Adding equal volumes of different electrolytes to the deuterated water D_2_O solvent and testing the ^1^H spectra. X-ray photoelectron spectroscopy (XPS) was characterized by Thermo Fisher ESCALAB Xi + with a monochromatic Al Kα X-ray source (energy resolution = 0.43 eV). X-ray diffraction (XRD) patterns were measured by the equipment (D8 ADVANCE A25) with Cu Kα radiation (*λ* = 0.15406 nm). The morphologies of samples were investigated by field emission scanning electron microscope (SEM, SNE-4500M Plus) and laser confocal scanning microscope (LCSM, LEXT-OLS4000).

### Electrochemical Measurement

All the cells were assembled using CR-2032-coin cases in an open-air environment and glass fiber filters (GF/D, Whatman) as separators. The electrolyte used in one cell was 100 μL. The symmetrical cells were assembled with Zn plate discs (thickness: 50 μm, *φ* = 12 mm). The asymmetrical cells were assembled with Zn plate discs (thickness: 50 μm, *φ* = 12 mm) as the anode and Cu plate discs (thickness: 50 μm, *φ* = 19 mm) as the cathode. A NEWARE battery tester was used to test the discharge/charge measurements of those cells. The linear polarization, linear sweep voltammetry (LSV), chronoamperograms (CA), and cyclic voltammetry (CV) were carried out on the electrochemical workstation (Autolab, Metrohm, PGSTAT204). Linear polarization curves were tested using Zn|Zn symmetrical cells by scanning between − 0.1 and 0.06 V at a scan rate of 1 mV s^−1^. LSV curves were tested using Zn|Zn symmetrical cells from − 0.1 to 0 V at 1 mV s^−1^. CA curves were tested under an overpotential of − 150 mV for 150 s. Zn|Cu asymmetrical coin cells were used to test CV curves of the Zn plating/stripping at a scan rate of 1 mV s^−1^.

### Density Functional Theory Calculation

In this work, under the scheme of generalized gradient approximation [32], with the use of PBE functional [33] and double numerical polarized (DNP) basis, as embedded in DMol3 package [34], the spin-polarized density functional theory (DFT) calculations were carried out. A global orbital cutoff with a radius of 4.5 Å was employed, under which the adsorption geometry was fully relaxed; while, the total energy and atomic force approached to 10^–6^ Ha and 0.005 Ha Å^−1^ (1 Ha ≈ 27.2114 eV). DNP basis, which is comparable in size to Gaussian basis 6–311 + G** sets [35], has been extensively tested and successfully used in surface adsorption of organic molecules [36]. An explicit model (COSMO, with a dielectric constant of 78.5, as the default in DMol3) was used to consider the solution effect. The Zn surface has been modeled with the slab for Zn(001)-p(4 × 4) for molecule adsorption, with a large vacuum space (> 15 Å along the z-axis). K-space has been sampled with Gamma point because of the large dimension size. Including the highest occupied and lowest unoccupied MO, frontier molecule orbitals (MO) have been obtained (labeled as HOMO and LUMO) and visualized with calculated eigenvalues. Adsorption energy ($E_{{{\text{ads}}}}$), $E_{{{\text{ads}}}} = E_{{\left( {{\text{A}} - {\text{S}}} \right)}} - E_{{\left( {\text{A}} \right)}} - E_{{\left( {\text{S}} \right)}}$, in which $E_{{\left( {\text{S}} \right)}}$, $E_{{\left( {\text{A}} \right)}}$ and $E_{{\left( {{\text{A}} - {\text{S}}} \right)}}$ are the calculated total energies of clean Zn(001) surface (S), adsorbed small molecules (A = water, Glu, Gly, His, Ser) and A-adsorbed on Zn(001). Under this scheme, the larger the negative Eads, the stronger the adsorption capacity.

### Force-Field Molecule Dynamics

To investigate the coordination of Zn^2+^ ions, force field molecular dynamics (MD) of Zn^2+^ in H_2_O solution, containing various ligands (*L*), including H_2_O, ${\text{SO}}_{4}^{{{2} - }}$, Glu, and His amino acids, has been carried out by using COMPASS III force field [37], which has been extensively tested in solvated metal ions [38, 39]. Initially tested as single Zn^2+^ dissolved in water solution, showing typical [Zn(H_2_O)_6_]^2+^ structure with a Zn–O distance of 2.02–2.10 Å. The Zn^2+^ solution was simulated with 20 Zn^2+^ and 20 ${\text{SO}}_{4}^{{{2} - }}$ dissolved in 555 water molecules. The MD simulations were performed with a time step Δ*t* = 1 fs, running for 5000 ps, in which the last 1000 ps have been employed for further analysis, such as Zn^2+^ diffusion and dissolved geometry. An NVT ensemble with *T* = 298.0 K was used for simulation, and the initial geometry was obtained from full optimization with energy converged to 10^–4^ kcal mol^−1^ and force converged to 0.005 kcal mol^−1^ Å^−1^. A Nose thermostat was operated to mediate the temperature. For MD simulations, 20 pairs of Zn^2+^–${\text{SO}}_{4}^{{{2} - }}$ were randomly distributed in water. Once the Zn^2+^ solution has been obtained from MD, a single amino acid (Glu and His) has been loaded and coordinated to one Zn^2+^, followed by independent MD simulations. The radial distribution function (RDF) and coordination number (CN) were calculated based on the collected final configuration.

### Multiphysics Simulation

COMSOL Multiphysics 6.2 software was used to build and solve the Multiphysics simulation model. In this work, we constructed a Zn metal anode surface with a certain surface roughness based on Gaussian random and uniform random distribution functions in COMSOL. The roughness of the electrode surface changing within the electrodeposition process was generated by: $f\left( {x,y} \right) = \sum\nolimits_{m = - M}^{M} {\mathop \sum \nolimits_{n = - N}^{N} } a\left( {m,n} \right){\text{cos}}\left[ {2\pi \left( {mx + ny} \right) + \varphi \left( {m,n} \right)} \right]$, where $x$ and $y$ are the spatial coordinates; $m$ and $n$ are the spatial frequencies; $a\left( {m,n} \right)$ is the amplitude and $\varphi \left( {m,n} \right)$ is the phase angle [40]. The model was built using ultrafine grid division, and the maximum grid size was 0.015 µm. Therefore, the 3D dynamic solution of the Zn-ion deposition process was carried out by using a deformed mesh function in COMSOL based on the simulated electric field, which is influenced by Faraday's laws of electrolysis, the Nernst–Einstein relationship and the classic Butler–Volmer equation $i = i_{0} \{ \exp (\frac{{\alpha_{a} zF}}{RT}\eta ) - \exp (\frac{{\alpha_{c} zF}}{RT}\eta )\}$, where $i_{0}$ is the exchange current density; $E$ is the electrode potential; $E_{eq}$ is the equilibrium potential; $\alpha_{a}$ and $\alpha_{c}$ are the charge transfer coefficient in the anodic and cathodic directions; $z$ is the number of electrons involved in the electrode reaction; $F$ and $R$ are Faraday constant and gas constant. The solution process was based on the MUMPS solver. The deposition efficiency was assumed to be 100% and the occurrence of any side reactions was not considered. The variance of simulated faradic current density and surface height over the entire surface were calculated using the formula: $s^{2} = 1/n\left[ {\left( {x_{1} - \overline{x}} \right)^{2} + \left( {x_{2} - \overline{x}} \right)^{2} + \cdots + \left( {x_{n} - \overline{x}} \right)^{2} } \right]$, where $x_{n}$ is the value of faradic current density or surface height of each data point, and $\overline{x}$ is the average value of them [41, 42].

## Results and Discussion

**Fig. 1:**
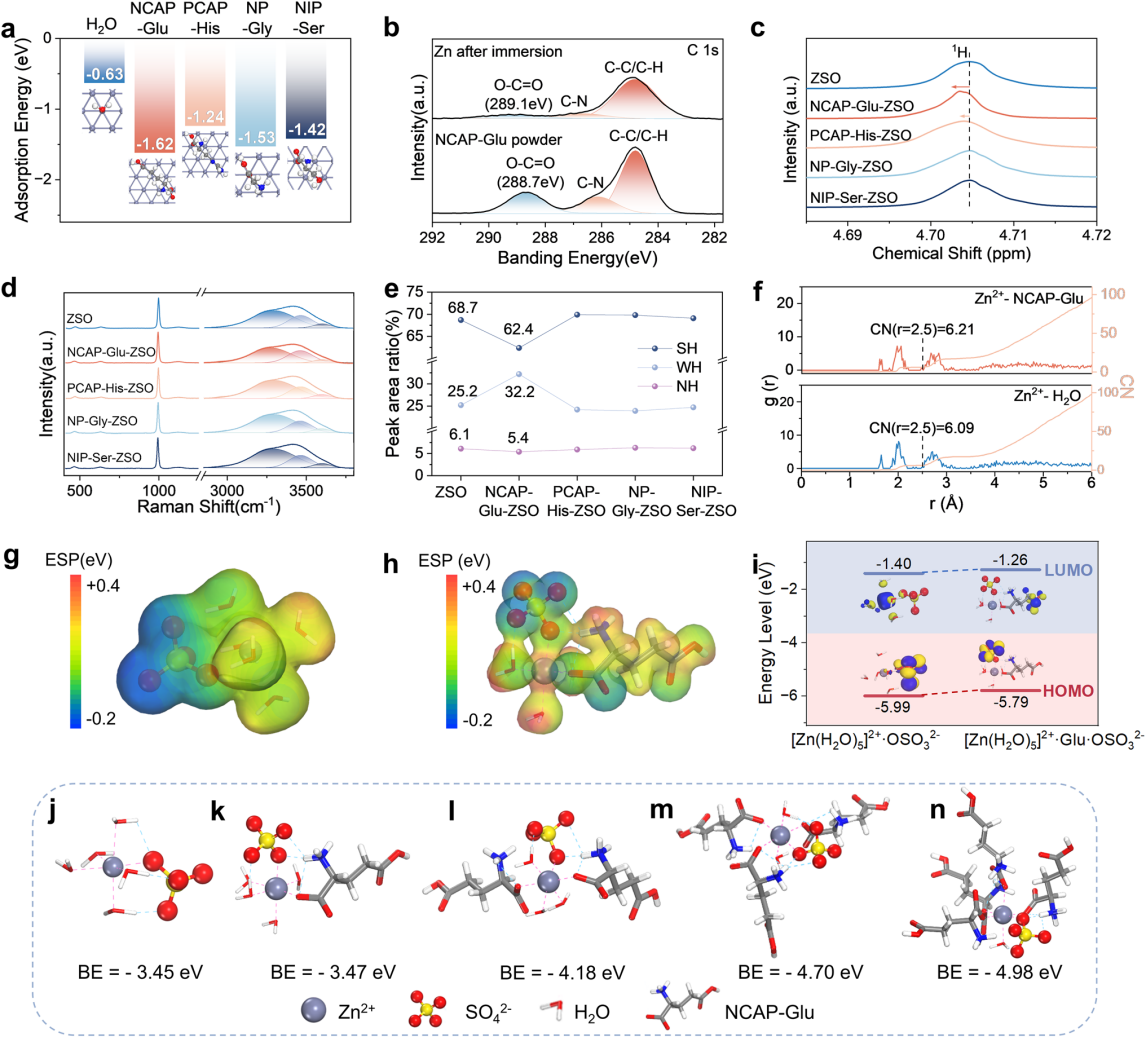
Hydrated structure reconstruction and adsorption effect from amino acid additives. **a** Adsorption energy of H_2_O, NCAP-Glu, PCAP-His, NP-Gly, and NIP-Ser molecules on Zn (002) surface. **b** XPS spectra of Zn metal after immersion and glutamate powder. **c** NMR spectra of different electrolytes. **d** Raman spectra of different electrolytes and the **e** area ratio of H–O peaks in Raman spectra. **f** Radial distribution functions and coordination numbers of Zn^2+^-NCAP-Glu and Zn^2+^–H_2_O in NCAP-Glu-ZSO. Electrostatic potential mapping of **g** [Zn(H_2_O)_5_]^2+^·${\text{SO}}_{4}^{{{2} - }}$ and **h** [Zn(H_2_O)_4_]^2+^·Glu·${\text{SO}}_{4}^{{{2} - }}$. **i** Highest occupied molecular orbital and lowest unoccupied molecular orbital of [Zn(H_2_O)_5_]^2+^·${\text{SO}}_{4}^{{{2} - }}$ and [Zn(H_2_O)_4_]^2+^·Glu·${\text{SO}}_{4}^{{{2} - }}$. Optimized geometry for local Zn^2+^ coordinated with ligands and the binding energy of [Zn(H_2_O)_x_]^2+^·Glu_y_·${\text{SO}}_{4}^{{{2} - }}$, **j** *x* = 5, *y* = 0; **k** *x* = 4, *y* = 1; **l** *x* = 3, *y* = 2; **m** *x* = 2, *y* = 3; **n** *x* = 1, *y* = 4

To verify the expected mechanism of amino acid additives on the regulation of EDL on the Zn anode surface, DFT was used to calculate the adsorption energy of H_2_O and several soluble amino acids on the Zn (002) face. The results show that amino acids have higher adsorption energy on Zn anode than H_2_O, especially the NCAP-Glu delivers the highest energies of − 1.62 eV, meaning that amino acids have much stronger interaction with Zn metal anode than H_2_O molecules (Fig. 1a). The competing adsorption of amino acid molecules ensures the replacement of H_2_O adsorbed on Zn anode with amino acids to form a H_2_O-poor IHP, which is expected to prevent HER side reaction. On the other hand, the higher adsorption energy between amino acids and Zn metal demonstrates the zincophilic nature, which can guide Zn^2+^ flux and promote homogenous nucleation of Zn to restrict dendrite growth [43]. Zincophilic amino acids consist of strong interaction with Zn metal and Zn^2+^, in which Zn metal has been verified by DFT calculating. Thus, the strong interaction between Zn^2+^ and Glu by Zn–COO^−^ bond is confirmed using XPS, which shows that the peak density of O−C=O in Glu decreases accompanied by a broadening of the peak after being added to 2 M ZnSO_4_ (ZSO) electrolyte (Fig. 1b).

Furthermore, to verify the coordination structure regulation of Zn^2+^, NMR and Raman spectra were characterized. ^1^H NMR spectra (Fig. 1c) show the solvation structure reconstruction of hydrated Zn^2+^, where ^1^H chemical shift changes from 4.705 ppm in ZSO electrolyte to 4.703 ppm in NCAP-Glu-ZSO electrolyte and 4.704 ppm in PCAP-His-ZSO electrolyte. While there is no significant shift in ZSO electrolytes with addition of NP-Gly and NIP-Ser. It means that the addition of Glu and His decreases the electron density of ^1^H effectively due to the release of free H_2_O bounded by Zn^2+^ and changes the solvation structure of Zn^2+^ due to their polarity [44]. The O–H bond stretching vibrations in H_2_O molecules can be reflected between 2900 and 3700 cm^−1^ in Raman spectra, which can be divided into strongly hydrogen bond (SH) at ~ 3200 cm^−1^, weakly hydrogen bond (WH) at ~ 3400 cm^−1^, and strongly non-hydrogen bond (NH) at ~ 3558 cm^−1^ (Fig. 1d) [45]. As shown in Fig. 1e, the Raman peaks area ratios of the above three types vibration indicate that the addition of Glu effectively decreases the SH fraction from 68.7% to 62.4% compared with other amino acid additives; while, the WH fraction increases from 25.2% in ZSO to 32.2% in NCAP-Glu-ZSO, illustrating that the strong hydrogen bonding network is significantly disrupted in EDL structure with Glu adding. Therefore, amino acids, especially Glu, can be verified as useful EDL regulators due to their strong interaction with Zn and Zn^2+^.

Furthermore, MD simulation was conducted to confirm the effect of amino acid additives on the regulation of the solvation structure of Zn^2+^, in which Glu was chosen as an example according to the previous characterization. The statistical results of MD simulation indicate that the primary solvation shell of Zn^2+^ in ZSO consists of five H_2_O molecules with one SO_4_^2−^ anion. With the addition of Glu, one of the H_2_O molecules surrounding Zn^2+^ is replaced by Glu, forming [Zn(H_2_O)_4_]^2+^·Glu·SO_4_^2−^. Following, the solvation structure of Zn^2+^ can be quantitatively revealed using RDFs and CN analysis from MD calculation. As shown in Fig. 1f, the primary solvation shell of Zn^2+^ located at 1.65 Å contributed by Zn–OH (H_2_O) and 1.61 Å contributed by Zn-Glu in NCAP-Glu-ZSO electrolyte, and the CN number at 2.5 Å are 6.09 of Zn–OH (H_2_O) and 6.21 of Zn-NCAP-Glu, respectively. The results mean that the NCAP-Glu molecules are more competitive than H_2_O and it does participate in the primary solvation shell of Zn^2+^. Besides, single strongly coordination can be observed through local analysis of CN curves (Fig. S2). To reveal the intrinsic driving force for the formation of different solvation structures, the electrostatic potential (ESP) of the solvation structure was analyzed by DFT calculation. As shown in Fig. 1g, h, compared with that in ZSO electrolyte, the distribution of negative charge in NCAP-Glu-ZSO electrolyte is more scattered, which indicates the higher stability of the solvation structure as [Zn(H_2_O)_4_]^2+^·Glu·SO_4_^2−^ [46]. Besides, the molecular orbitals (Fig. 1i) of different solvation structures calculated by DFT can reflect their electrochemical stability. The LOMO energy level of [Zn(H_2_O)_4_]^2+^·Glu·SO_4_^2−^ is − 1.26 eV, which is higher than − 1.40 eV of [Zn(H_2_O)_5_]^2+^·SO_4_^2−^, confirming the higher reduction stability of NCAP-Glu-ZSO. Therefore, the side reaction of HER on the surface of Zn metal could be suppressed by H_2_O replacing inside the primary solvation shell with Glu molecules. Then, more Glu molecules are added into the primary solvation shell gradually via thermodynamics. As shown in Fig. 1j–n**,** with Glu replacing H_2_O molecules one by one, the binding energy of solvated Zn^2+^ shifts to a higher level. Typically, the binding energy of [Zn(H_2_O)_3_]^2+^·2Glu·SO_4_^2−^ is − 4.18 eV, and it increases sharply when one more Glu replaces one H_2_O to form [Zn(H_2_O)_2_]^2+^·3Glu·SO_4_^2−^ of − 4.7 eV, while it ups slowly to add one more Glu to form [Zn(H_2_O)]^2+^·4Glu·SO_4_^2−^ (− 4.98 eV), which demonstrates hydrated Zn^2+^ becomes more stable as H_2_O molecules are replaced and only 4 Glu molecules can squeeze into the Zn^2+^ primary solvation shell. Therefore, it is verified that the zincophilic amino acids, especially Glu with NCAP characteristic, ensure preferentially adsorption on the Zn metal anode to regulate the EDL structure and the reconstruction of hydrated Zn^2+^ clusters. Therefore, dendrites growth, HER, and side reactions can be suppressed effectively.

**Fig. 2:**
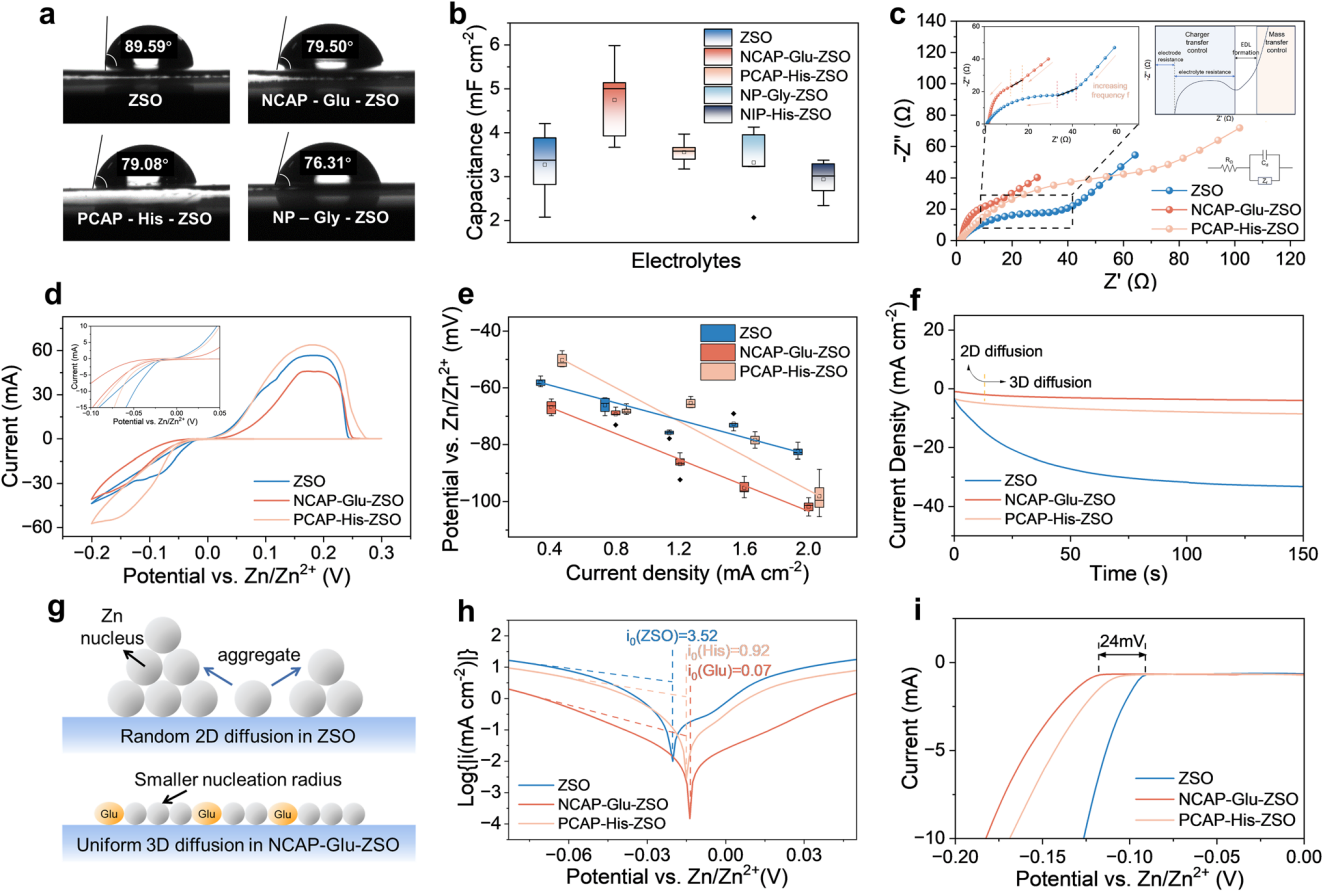
Interface behavior on Zn anodes surface of different electrolytes. **a** Wetting angle of different electrolytes on Zn anodes. **b** EDL capacitance on Zn anode surface in different electrolytes. **c** Impedance of Zn|Zn symmetrical cells using different electrolytes. **d** Cyclic voltammetry curves of Zn|Cu asymmetrical batteries with/without amino acids. **e** Nucleation overpotential on Zn anode surface at different current densities in different electrolytes. **f** Chronoamperograms of Zn metal in different electrolytes. **g** Diagram of nucleation and growth of Zn metal surface in electrolytes with/without glutamate adding. **h** Tafel curves of Zn|Zn symmetrical cells in different electrolytes. **i** Linear sweep voltammetry curves of Zn anode in different electrolytes

To explore the interface behaviors on the Zn anode, wetting angles of different electrolytes on the Zn anode were tested. Besides the negligible increment of the wetting angle between Zn metal and electrolyte with the addition of Ser, the electrolytes with the addition of Glu, Gly, and His show few decrements in wetting angles against Zn metal anode (Figs. 2a and S3). The smaller wetting angles of the electrolytes with additives indicate stronger interaction with the Zn metal anode, which foreshadows superior Zn-ion diffusion dynamics in EDL. Furthermore, EDL capacitance in different electrolytes were calculated to verify the effect of the addition of amino acids on the regulation of EDL structure. CV test using different scan rates ranging from 2 to 16 mV s^−1^ was conducted; and thus, the capacitance of EDL was obtained (Figs. S4 and S5). As shown in Fig. 2b, EDL on the Zn anode with the electrolyte of NCAP-Glu-ZSO is enhanced than that with ZSO; while, those with the rest of electrolytes show changeless or negligible lowering. Thus, compared to other amino acids, the addition of Glu results in the increment of EDL significantly due to its NCAP characteristic with strong zincophilic, indicating the improved transportation dynamics of Zn^2+^ on the anode surface. Furthermore, the regulation of EDL on Zn anodes was verified via the chemical impedance. As shown in Figs. 2c and S6, the resistance decreases with adding Glu compared with others. Moreover, the slope of the internal liner range of EIS spectra is lowered with adding Glu, confirming that diffusion dynamics of Zn^2+^ are improved in EDL with the addition of Glu [47].

Following, the effect of EDL improvement on the electro-crystallization including nucleation and growth of Zn metal in different electrolytes were further investigated. The CV test was applied to evaluate the delay of the cathodic current. As shown in Figs. 2d and S7, the decreased cathodic current in NCAP-Glu-ZSO electrolyte is caused by the formation of polar groups-rich protective layer (–NH_2_ and –COOH in amino acids) on Zn anodes due to the preferentially adsorbed amino acids. Therefore, homogeneous nucleation and smaller nucleation radius are obtained to ensure uniform Zn deposition on the anode. When a series of current densities from 0.4 to 2.0 mA cm^−2^ were applied, the slope of nucleating overpotentials was upped with the addition of amino acids, which demonstrates that the nucleation process of Zn is significantly dominated by the additives through the regulation of EDL. In detail, the nucleating overpotential of Zn metal with the ZSO electrolyte without additives, increases with the addition of Glu while that decreases with the rest of the amino acids besides His (Figs. 2e and S8). Based on the classical nucleation theory, the Gibbs free energy change of nucleation ($\Delta G_{nuc}$) of Zn atoms can be described as:

1 $$\Delta G_{{{\text{nuc}}}} = - \frac{2\pi }{3}r^{3} \frac{{\Delta G_{{\text{B}}} }}{{V_{{\text{m}}} }} + 2\pi r^{2} \sigma$$

where $r$ is the radius of semi-sphere nuclei; $\Delta G_{{\text{B}}}$ is the Gibbs free energy change when a Zn atom is adsorbed to the surface compared to being in the supersaturated electrolyte; $V_{{\text{m}}}$ is the molar volume of Zn metal; $\sigma$ is the surface tension between Zn nuclei and the electrolyte. In the electrochemical system, $\Delta G_{{\text{B}}}$ is related to the overpotential (*η*) through

2 $$\Delta G_{{\text{B}}} = - nF\left| {\upeta } \right|$$

Substituting Eq. (2) into Eq. (1) and take the derivative of the radius of $\Delta G_{{{\text{nuc}}}}$ [48], the critical radius of nuclei can be described as:

3 $$r_{{\text{c}}} = \frac{{2\sigma V_{{\text{m}}} }}{{F\left| {\upeta } \right|}}$$

Based on Eq. (3) that derived from the classical nucleation theory, the higher the nucleation overpotential, the smaller the critical nucleation radius of the nuclei. Thus, the fine critical radius of nuclei can be expected when adding NCAP-Glu as additives and a larger critical radius of nuclei can be expected when adding NP-Gly and NIP-Ser [49]. In terms of His, its critical nucleation size change depends on the applied current density, with lower overpotential to form a larger Zn nucleus at a small current density while higher overpotential to form a smaller Zn nucleus at a higher current density [50]. Following, an overpotential of − 150 mV was applied to the Zn anode to test CA curves. As shown in Figs. 2f and S9, in the bare ZSO electrolyte, the anode's current density increases continuously, indicating prolonged 2D diffusion. In comparison, with amino acids added, the current density tends to become stable immediately, especially in NCAP-Glu-ZSO electrolyte, which verifies the transformation from 2D diffusion to 3D diffusion. A larger nucleus is easy to develop into a dendritic formation combined with random 2D diffusion. When adding Glu to reinforce EDL on the Zn anode surface, the dense and dendrite-free electrodepositing morphology of Zn metal can be expected by the fine nucleus with uniform 3D diffusion (Fig. 2g).

Besides the electrodeposition of Zn metal that is dominated by the EDL structure, the surface side reaction of HER and self-corrosion are also controlled by the regulated EDL structure. Tafel curves of Zn|Zn symmetrical cells were characterized to calculate corrosion current density and corrosion potential of Zn anode using a series of electrolytes with various amino acid additives. As shown in Fig. S10, the left-wing of a typical Tafel curve represents the self-corrosion of Zn in the gentle acidic electrolyte ${\text{Zn}} \leftrightharpoons {\text{Zn}}^{2 + } + 2e^{ - }$; while, the right-wing represents the combination of HER reaction of $2{\text{H}}^{ + } + 2e^{ - } \leftrightharpoons {\text{H}}_{2} \uparrow$ with the depolarization reaction of ${\text{Zn}}^{2 + } + 2e^{ - } \leftrightharpoons {\text{Zn}}$ [51]. As shown in Figs. 2h and S11, the corrosion current density of Zn anode in the electrolytes with amino acids’ adding is significantly decreasing than that in ZSO electrolyte, revealing that self-corrosion on Zn metal can be suppressed with the amino acid additives. In particular, Glu embraces the greatest anti-self-corrosion effect among others. Furthermore, LSV was characterized to verify the ability of amino acids to inhibit the side reaction of HER. As shown in Figs. 2i and S12, the initial potential for HER increases ~ 24 mV in the electrolyte with Glu additive, which indicates Glu can effectively suppress the HER side reaction. Besides, the other amino acids also up the initial potential for HER more or less. It can be illustrated that the introduction of Glu can significantly reduce the free H_2_O molecules in IHP and suppress H_2_O adsorption on the Zn anode surface to restrain HER. In conclusion, the polarity of Glu and His could influence the charge distribution around H^+^, thus changing the solvation structure of the hydrated Zn^2+^ in OHP. Besides, acidic Glu shows the best EDL regulation ability because it is negative-charged after ionization, which guarantees strong interaction with Zn^2+^/Zn. Therefore, the NCAP characteristic of Glu makes it useful as EDL regulator.

**Fig. 3:**
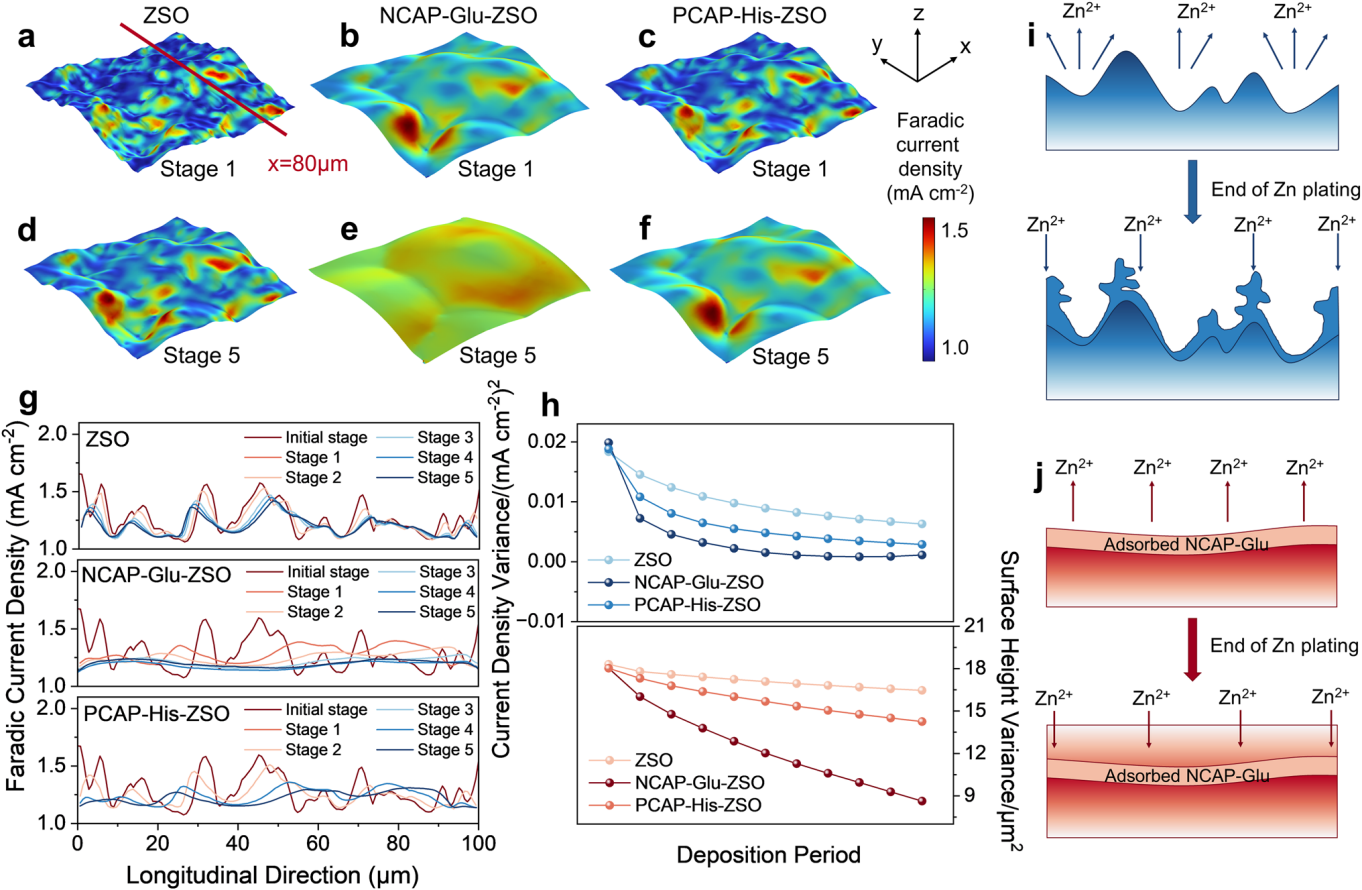
Multiphysics simulation of current density distribution on Zn anode surface after 1/5 deposition process in **a** ZSO electrolyte; **b** NCAP-Glu-ZSO electrolyte; **c** PCAP-His-ZSO electrolyte; and after an entire deposition process in **d** ZSO electrolyte;** e** NCAP-Glu-ZSO electrolyte; **f** PCAP-His-ZSO electrolyte at 1 mA cm^−2^ with a capacity of 1 mAh cm^−2^. **g** Longitudinal current density distribution of Zn anode surface in different electrolytes at the location of *x* = 80 μm. **h** Variance of surface Faradic current density and surface height of Zn anode in the selected area (100 μm × 100 μm) during an entire deposition process in different electrolytes. Schematic diagram of simulated Zn^2+^ plating/stripping in **i** ZSO electrolyte; **j** NCAP-Glu-ZSO electrolyte

To further testify the dendrite suppression ability of NCAP-Glu-ZSO electrolyte with the regulation of EDL, Multiphysics simulation was used to simulate the evolution of Zn^2+^ concentration and corresponding current density distribution on dynamically evolving Zn anode surface. Based on DFT calculation and the interfacial behavior of different electrolytes, Glu and His, were selected due to their preferentially adsorption on the Zn anode and the ability to reconstruct the solvation structure of hydrated Zn-ions. Before the simulation, a random 3D surface with a roughness of 18.85 μm was generated, which size is 100 μm × 100 μm (Fig. S13). Due to the regulation of EDL on the surface with Glu or His additives, the Faradic current density distributes variously on the 3D surface. As shown in Fig. 3a, Faradic current density is mainly concentrated at the bulges on the random surface with ZSO electrolyte at the first stage, while the bare appearance of Faradic current density at the valley. The distribution of Zn^2+^ on the surface is consistent with that of the current density (Fig. S14). With the addition of Glu as an additive, the Faradic current density distributes relatively uniformly on the substrate with regulated EDL, and the associated Zn^2+^ concentration is also even (Figs. 3b and S15). Whereas, the addition of His exhibits a negligible effect on the surface none a uniform electric field with the concentration of Zn^2+^ at the beginning stage (Figs. 3c and S16). As the electrodeposition of Zn processing, Faradic current density distribution on the Zn anode surface remains nonuniform, and obvious concentrated high current density areas appear at the bulges with the bare electrolyte of ZSO (Fig. 3d). With amino acids, the electric field uniformity of Zn anode becomes more even in NCAP-Glu-ZSO electrolyte with Zn’s electrodepositing (Fig. 3e); while, the electric field of Zn anode in PCAP-His-ZSO is more even than that in ZSO electrolyte, whereas not as uniform as in NCAP-Glu-ZSO (Fig. 3f). Moreover, as the result of the uniform distribution of Faradic current density as well as the concentration of Zn^2+^ on the surface, the initial rough surfaces with NCAP-Glu-ZSO and PCAP-His-ZSO electrolytes become more uniform electrodeposition morphology (Fig. 3d–f).

To analyze the evenness of Faradic current density on the Zn anode surface and associated surface geometrical evolution, several position-fixed longitudinal areas at the locations of *x* = 20, 40, 60, 80 μm (Figs. 3a and S17–S20) were selected to analyze the evenness of the electric field on the Zn anode surface, respectively. As shown in Figs. 3g and S18–S20, the changing of the surface electric field during the whole electrodeposition process can be observed. As the electrodeposition is processed, the electric field on the Zn anode in the NCAP-Glu-ZSO electrolyte gradually becomes more even. Oppositely, it remains uneven in the ZSO electrolyte during the entire Zn^2+^ electrodeposition process. In PCAP-His-ZSO electrolyte, the electric field uniformity is improved, while not as good as that in NCAP-Glu-ZSO. Variance could reflect the deviation of a set of data and indicate the volatility. The variance of simulated faradic current densities and surface heights data over the entire surface were calculated. As shown in Fig. 3h, the variance of Faradic current density decreases with electrodeposition of Zn metal, and that with NCAP-Glu-ZSO is the smallest among others, illustrating that the process of electrodeposition, the electrochemical reaction of ${\text{Zn}}^{2 + } + 2e^{ - } \leftrightharpoons {\text{Zn}}$, on Zn anode using NCAP-Glu-ZSO electrolyte, becomes more even. Correspondingly, the height fluctuation of the Zn anode surface supports the above results, the Zn anode surface becomes more uniform after deposition in NCAP-Glu-ZSO than that in the ZSO electrolyte. The analysis of simulated data further confirms that the addition of Glu could uniform the electric field on the Zn anode, to even the Zn^2+^ electrodeposition and uniform the Zn anode surface (Fig. 3i, j).

**Fig. 4:**
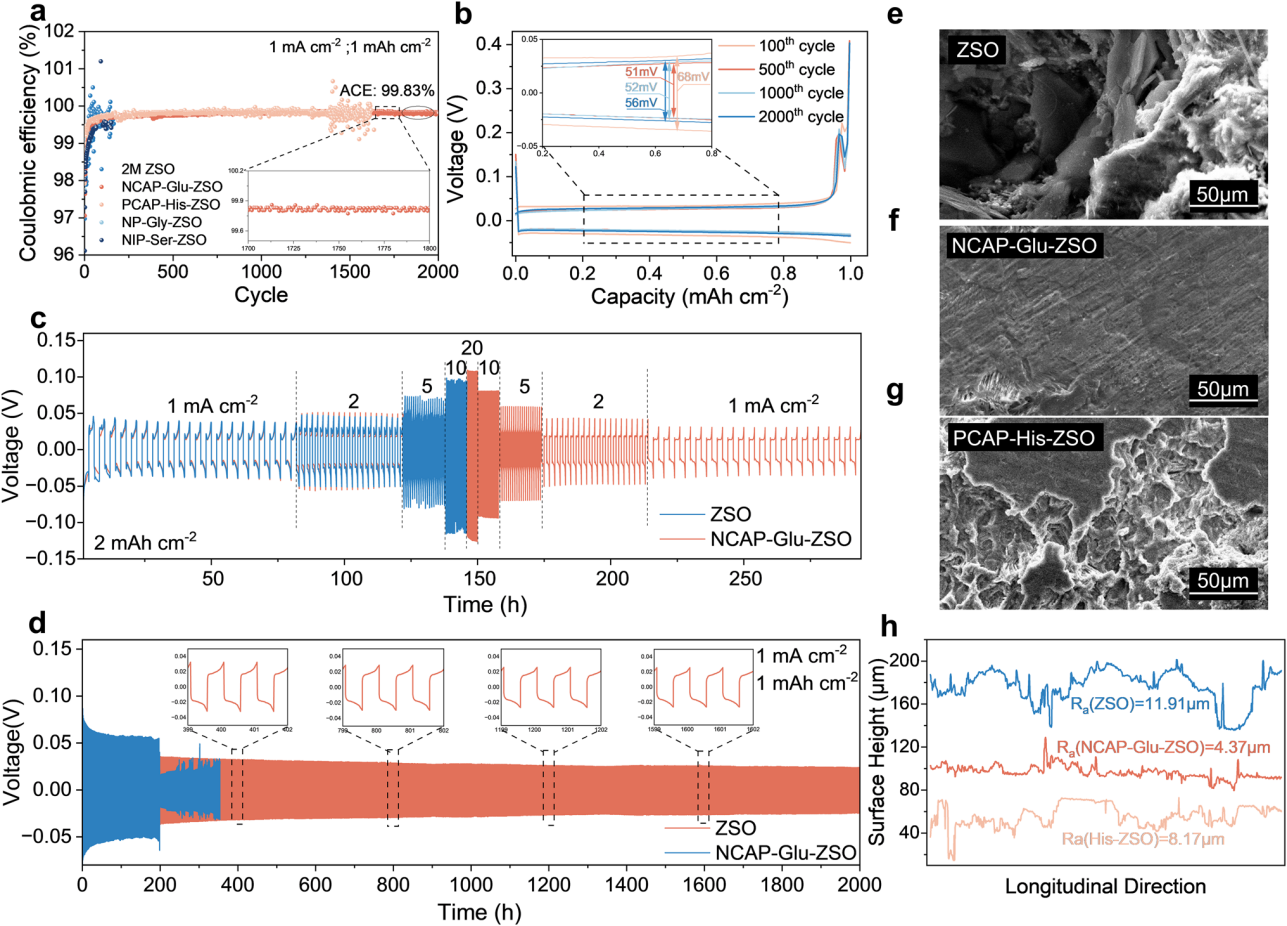
Electrochemical reversibility and stability of Zn metal anode. **a** Coulombic efficiency of Zn|Cu cells at 1 mA cm^−2^ and **b** corresponding capacity–voltage curves at different cycles. Voltage–time profiles of Zn|Zn symmetrical cells **c** changing the current density from 1 to 20 mA cm^−2^ and **d** that at a current density of 1 mA cm^−2^ with a capacity of 1 mAh cm^−2^. SEM images of cycled Zn metal anode after 50 cycles in different electrolytes of **e** ZSO; **f** NCAP-Glu-ZSO; **g** PCAP-His-ZSO. **h** Two-dimensional height fluctuation of Zn anode surface after 50 cycles in different electrolytes

Based on the detailed theoretical and experimental analyses, the uniform interface of Zn metal anode with suppressed HER side reaction, anti-self-corrosion and tightly electrodeposited morphology can be guaranteed by the introduction of Glu as an EDL regulator, which foreshadows high electrochemical stability of Zn metal. Zn|Cu asymmetrical cells were assembled to evaluate the reversibility of Zn plating/stripping in different electrolytes. Consistent with the results of previous characterization, at a low areal capacity of 1 mAh cm^−2^, the Zn|Cu cell using NCAP-Glu-ZSO electrolyte can run stably more than 2000 cycles with an ultra-high average Coulombic efficiency (CE) of 99.83%, while the reference cell using ZSO electrolyte fails quickly after about 200 cycles and the CE fluctuates drastically (Fig. 4a). The performance of Zn plating/stripping with the electrolyte using His is also relatively improved while not as well as that using Glu, which results from their opposite charges after ionization. Negative charged Glu guarantees a strong interaction with Zn^2+^ to achieve better reversibility. Besides, as shown in Fig. 4b, the capacity–voltage curve of the Zn|Cu cell using NCAP-Glu-ZSO electrolyte indicates excellent reversibility of charging and discharging, unveiling the good reversibility of Zn plating/stripping. The stability of the Zn anode during plating/stripping was tested in Zn|Zn symmetrical cells. As shown in Fig. 4c, the symmetrical cell using NCAP-Glu-ZSO electrolyte exhibits stable cycling performance for more than 2000 h at a current density of 1 mA cm^−2^, in which steady and smooth voltage profiles are performed with a stable polarization voltage of about 25 mV. On the contrary, the Zn|Zn cell with the bare electrolyte of ZSO fails after ~ 200 cycles due to a short circuit. Furthermore, Zn|Zn symmetrical cells were tested at various current densities ranging from 1 to 20 mA cm^−2^ with a constant capacity of 2 mAh cm^−2^ to confirm the rate stability of Zn anode in NCAP-Glu-ZSO (Fig. 4d). During the entire cycles, the cell using NCAP-Glu-ZSO electrolyte exhibits great performance with stable voltage profiles and reversibility under each current density, which is much superior to that using ZSO electrolyte. It is confirmed that the addition of NCAP-Glu in electrolytes significantly increases the stability and reversibility of the Zn anode during the plating/stripping processes.

Based on the above results, NCAP-Glu performs the best among the chosen amino acids, and then PCAP-His. Therefore, the Glu and His were chosen as electrolyte additives to visually verify the dendrite and corrosion suppression abilities by field emission SEM images of Zn anodes after cycling. As shown in Fig. S21, after 10 cycles at a current density of 1 mA cm^−2^, the Zn anode surface in ZSO electrolyte tends to be uneven; while, aggregation of metallic Zn clusters is observed while holes caused by corrosion appear. On the contrary, the Zn anode surface in the NCAP-Glu-ZSO electrolyte is smooth with no dendrite growth. However, although without dendrites growth, an uneven surface can be observed on the Zn anode in the PCAP-His-ZSO electrolyte. After 50 cycles (Fig. 4e–g), severe dendrites and obvious corrosion are observed on the Zn anode in the ZSO electrolyte with disordered growth of Zn-hexagonal crystals. In contrast, the anode surface in NCAP-Glu-ZSO electrolyte still maintains uniform and smooth, which confirms the superior suppression abilities of dendrite growth and corrosion. As for the Zn anode in PCAP-His-ZSO electrolyte, despite obvious corrosion, there exists no aggregated dendrite growth. Furthermore, LCSM images were investigated to evaluate the surface flatness of the Zn anode after cycling visually. As shown in Fig. 4h, the 2-D Zn anode height fluctuation in NCAP-Glu-ZSO is much smoother than that in the ZSO electrolyte. After randomly selecting regions on the Zn anode surface, the roughness of Zn anodes in modified electrolytes, especially in NCAP-Glu-ZSO, exhibits much lower roughness compared with that in ZSO after 50 cycles. The results further confirm the stability and reversibility of Zn plating/stripping with Glu addition.

**Fig. 5:**
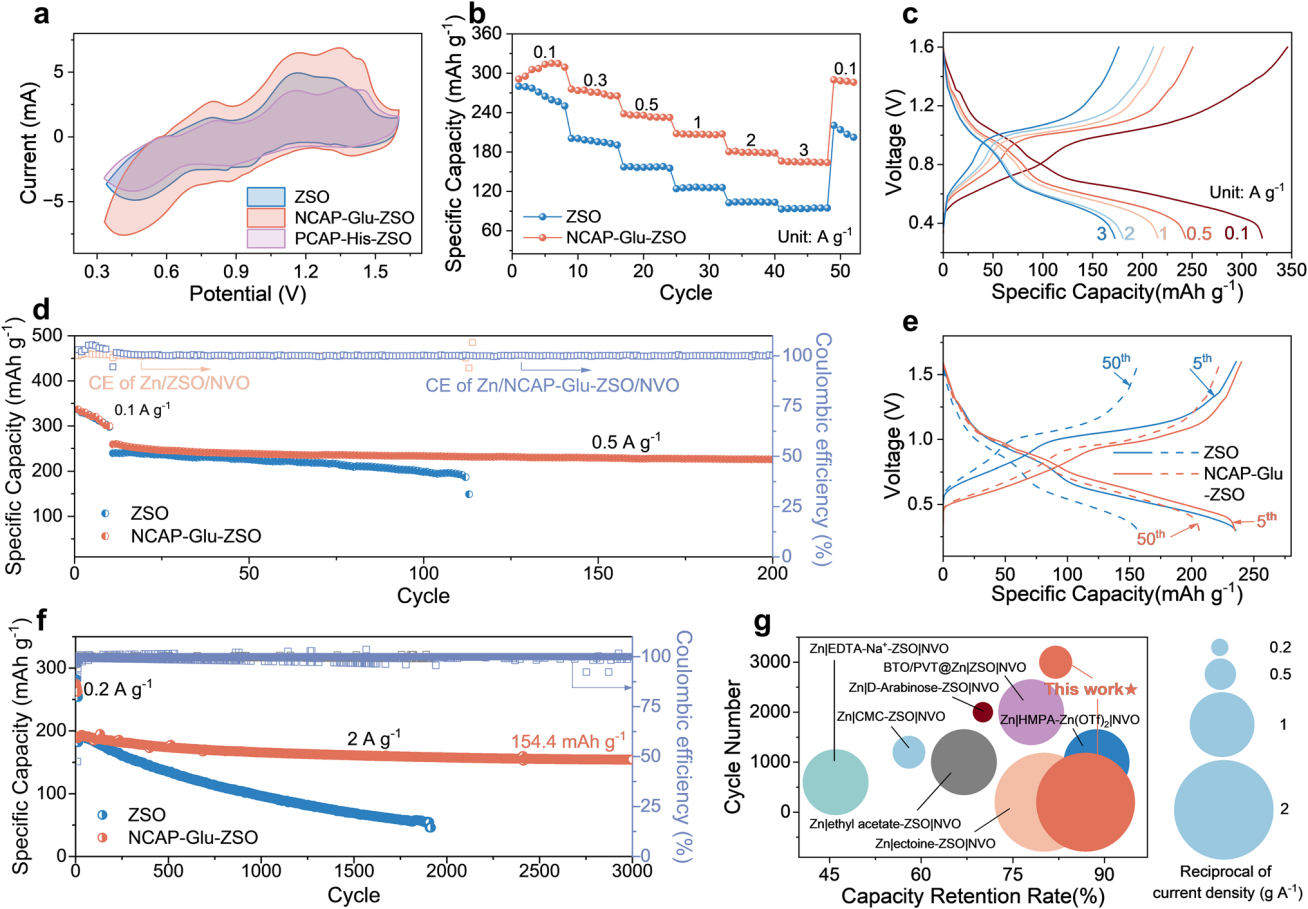
Electrochemical performance of NH_4_V_4_O_10_|Zn full cells. **a** Cyclic voltammetry curves of NH_4_V_4_O_10_|Zn full cells with/without glutamate at a scan rate of 0.1 mV s^−1^. **b** Rate capability of NH_4_V_4_O_10_|Zn cells at various current densities from 0.1 to 3 A g^−1^, and **c** corresponding voltage profiles of different current densities with/without glutamate. Cycling performance of NH_4_V_4_O_10_|Zn cells at a current density of **d** 0.5 A g^−1^, and **e** corresponding voltage profiles of different cycles with/without glutamate. **f** Cycling performance of NH_4_V_4_O_10_|Zn cells at a current density of 2 A g^−1^. **g** Comparison of the cycling performance of NH_4_V_4_O_10_|Zn full cells in this work with those reported ones in the literature [25–27, 52–54]

To indicate the feasibility of the electrolyte with Glu added for practical AZIBs application, the NVO (Fig. S22) cathode was prepared to couple with Zn metal anodes and assemble full cells. The CV curves (Fig. 5a) reveal the same multiple pairs of oxidation and reduction peaks using electrolytes with/without NCAP-Glu added, which indicates that Glu shows barely effect on the insertion and extraction of Zn^2+^ inside NVO cathode. Besides, the CV curve of the cell using NCAP-Glu-ZSO electrolyte demonstrates a higher area than that using ZSO, which indicates higher capacitance due to the EDL regulation on the Zn anode. To verify the rate capability of full cells with Glu added, the current density was changed from 0.1 to 3 A g^−1^ step by step and then returned to 0.1 A g^−1^. As shown in Figs. 5b, c, and S23, at 3 A g^−1^, the capacity retention is 57.1% and then rises to 96.9% when the current density comes back to 0.1 A g^−1^; while, the capacity retention of the cell using ZSO electrolyte is only 78.8% after returning to a lower current density of 0.1 A g^−1^. Besides, the high-reversibility of capacity–voltage curves of different states with various current densities verifies the stability of NVO|Zn full cells. At a low current density of 0.5 A g^−1^ (Fig. 5d), NVO|Zn full cell with ZSO electrolyte fails after around 100 cycles; while, the cell using NCAP-Glu-ZSO electrolyte still runs stably after 200 cycles. The capacity–voltage curves at the 5th and 50th cycles are selected to analyze the reversibility of NVO|Zn full cells (Fig. 5e). The capacity of NVO|Zn cell using ZSO electrolyte decreases obviously; while, the discharge/charge capacity curves of the cell with Glu added are almost overlapping. As shown in Figs. 5f and S24, at a higher current density of 2 A g^−1^, the specific capacity of the full cell using NCAP-Glu-ZSO exhibits great stability. After long-term cycling of 3000 cycles, the capacity retention is up to 82.1% with a remaining capacity of 154.4 mAh g^−1^ and the average of CE is up to 99.8% for 3000 cycles. In comparison, the full cell using ZSO electrolyte fails after ~ 1800 cycles with a capacity retention of 25.2%. Compared with the NVO|Zn full cells that have been reported in literatures (Fig. 5g), our work shows favorable comprehensive cycling performance both at high and low current densities. Therefore, it is proved that NCAP-Glu could stabilize Zn anode as EDL regulators and be used as promising additives in practical applications.

## Conclusions

In summary, we introduced several kinds of amino acids with different characteristics into electrolytes to regulate EDL structure on the Zn anode interface. According to computational simulation and experimental results, amino acids, especially NCAP-Glu can adsorb on the Zn anode to form an H_2_O-poor IHP structure due to the polar functional groups, which induce the uniform transport of Zn^2+^ flux by electrostatic attraction. It is verified that NCAP-Glu additives could improve ion diffusion and nucleation dynamics on the Zn interface, thus achieving uniform Zn electrodeposition. Besides, NCAP-Glu can change the hydrated Zn^2+^ construction by replacing H_2_O molecules and reducing HER side reactions. The synergistic effect of NCAP-Glu significantly increases the stability and reversibility of the Zn anode during the plating/stripping processes. Zn|Cu asymmetrical battery using NCAP additives could achieve a reversible long-term cycle for more than 2000 cycles with an ultra-high average CE of 99.83%. The capacity retention of assembled NVO|Zn full cells at a current density of 2 A g^−1^ is up to 82.1% after long-term cycling of 3000 cycles, and the feasibility of NCAP-Glu as electrolyte additives for practical application is verified. In a nutshell, this NCAP principle in this contribution can be accepted as an effective guideline for selecting novel electrolyte additives for AZIBs.

## Supplementary Information

Below is the link to the electronic supplementary material.

Supplementary file1 (PDF 2549 kb)
