# Supplementary material for: Selection of Negative Charged Acidic Polar Additives to Regulate Electric Double Layer for Stable Zinc Ion Battery
Source: Nanomicro Lett. 2024 Aug 14;16:270. doi: 10.1007/s40820-024-01475-5 (PMC11324644; doi:10.1007/s40820-024-01475-5)
Supplement: Supplementary file 1 — Supplementary file1 (PDF 2549 kb) [file 40820_2024_1475_MOESM1_ESM.pdf]

Supporting Information for

Selection of Negative-Charged Acidic Polar Additives to Regulate Electric Double Layer for Stable Zinc Ion Battery

Xing Fan[1,#], Lina Chen[2,#], Yongjing Wang[1], Xieyu Xu[1], Xingxing Jiao[3], Peng Zhou[4], Yangyang Liu[1, 5, *], Zhongxiao Song[1, *], and Jiang Zhou[6, *]

[1] State Key Laboratory for Mechanical Behavior of Materials, Xi'an Jiaotong University, Xi'an 710049, P. R. China

[2] School of Materials Science and Engineering, Harbin Institute of Technology (Shenzhen), Shenzhen 518055, P. R. China

[3] Research Institute of Frontier Science, Southwest Jiaotong University, Chengdu 610031, P. R. China

[4] Hunan Provincial Key Defense Laboratory of High Temperature Wear-Resisting Materials and Preparation Technology, Hunan University of Science and Technology, Xiangtan 411201, P. R. China

[5] School of Instrument Science and Technology, Xi'an Jiaotong University, Xi'an 710049, P. R. China

[6] School of Materials Science and Engineering, Hunan Provincial Key Laboratory of Electronic Packaging and Advanced Functional Materials, Central South University, Changsha 410083, P. R. China

[#] Xing Fan and Lina Chen have contributed equally to this work.

*Corresponding authors. E-mail: liuyy0510@hotmail.com (Yangyang Liu); zhongxiaosong@xjtu.edu.cn (Zhongxiao Song); zhou_jiang@csu.edu.cn (Jiang Zhou)

Supplementary Table and Figures

Table S1 Classification of typical amino acids

Classification	Amino acids			
Negative-charged acidic polar amino acids	Aspartate	**Glutamate**		
Positive-charged alkaline polar amino acids	Lysine	Arginine	**Histidine**	
Non-Polar amino acids	**Glycine**	Alanine	Valine	Leucine
	phenylalanine	Proline	Isoleucine	
Non-ionic polar amino acids	**Serine**	Glutamine	Threonine	Cysteine
	Asparagine	Tyrosine		

Negative-charged acidic polar glutamate (NCAP-Glu), positive-charged alkaline polar histidine (PCAP-His), non-polar glycine (NP-Gly), and non-ionic polar serine (NIP-Ser) were selected based on water solubility and commonness, in which "acidic" means it is negative-charged after ionization and "alkaline" means it is positive-charged after ionization.

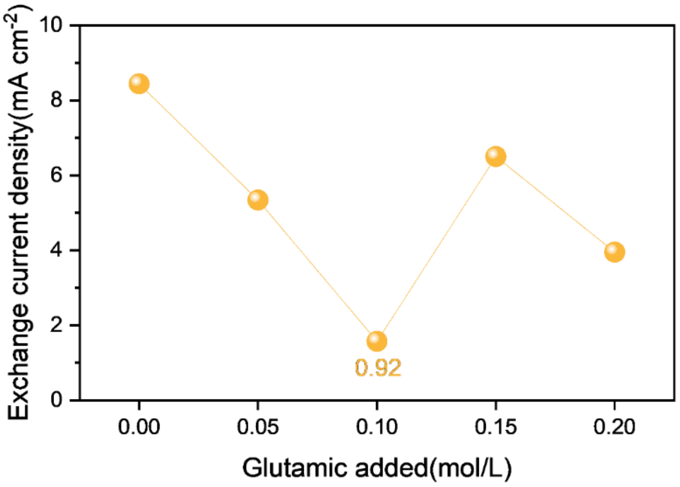

Fig. S1 Exchange current density on Zn anode surface with different concentrations of glutamate added

By comparison, the additive concentration of 0.1mol/L makes the electrode surface exchange current density the lowest, which ensures the most uniform nucleation. Therefore, the default concentration of additives was 0.1mol/L in this paper.

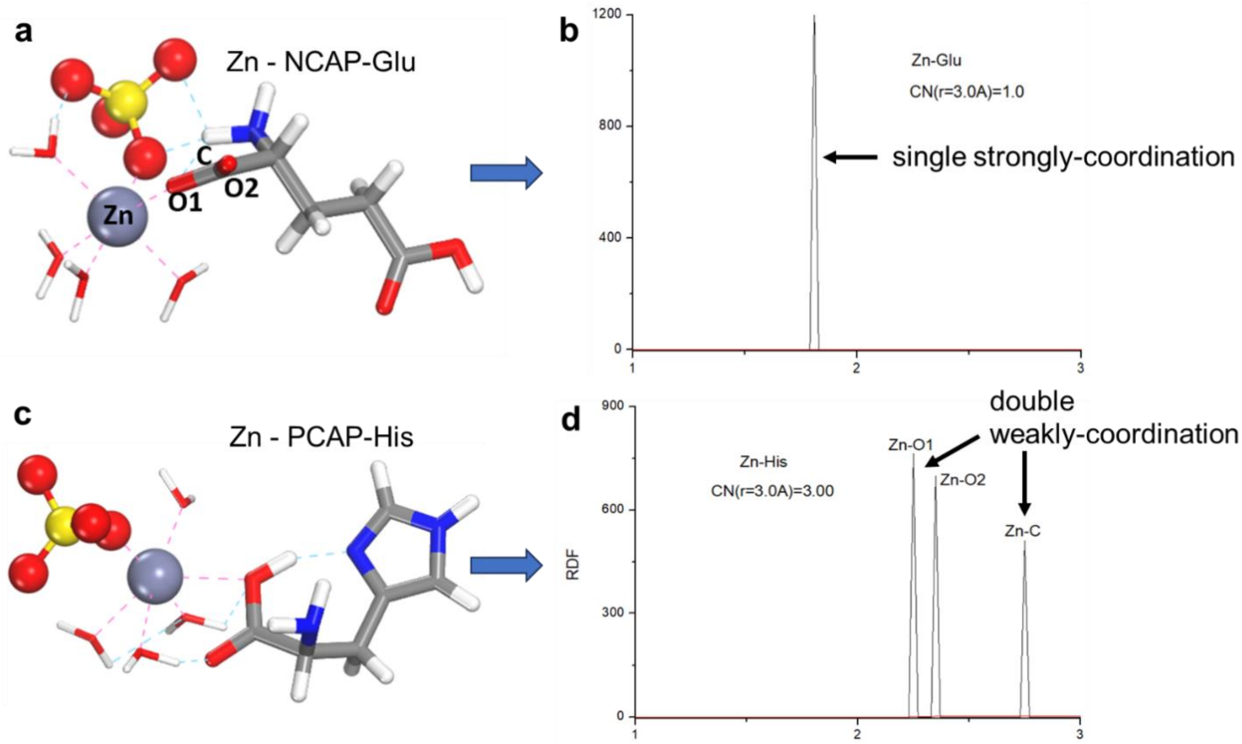

Fig. S2 MD Simulation of **a** coordination structure and **b** radial distribution of Zn^{2+}-NCAP-Glu in $[Zn(H_2O)_4]^{2+}$·Glu·SO_4^{2-}; **c** coordination structure and **d** radial distribution of Zn^{2+}- PCAP-His in $[Zn(H_2O)_4]^{2+}$·His·SO_4^{2-}

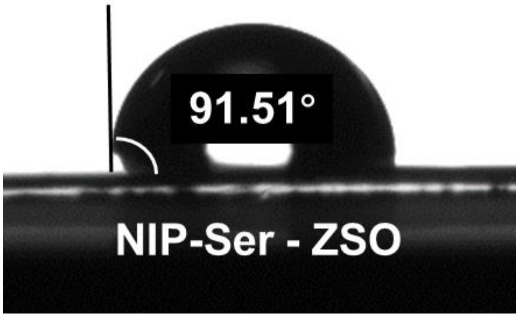

Fig. S3 Wetting angles of Ser-ZSO electrolyte on the Zn anode

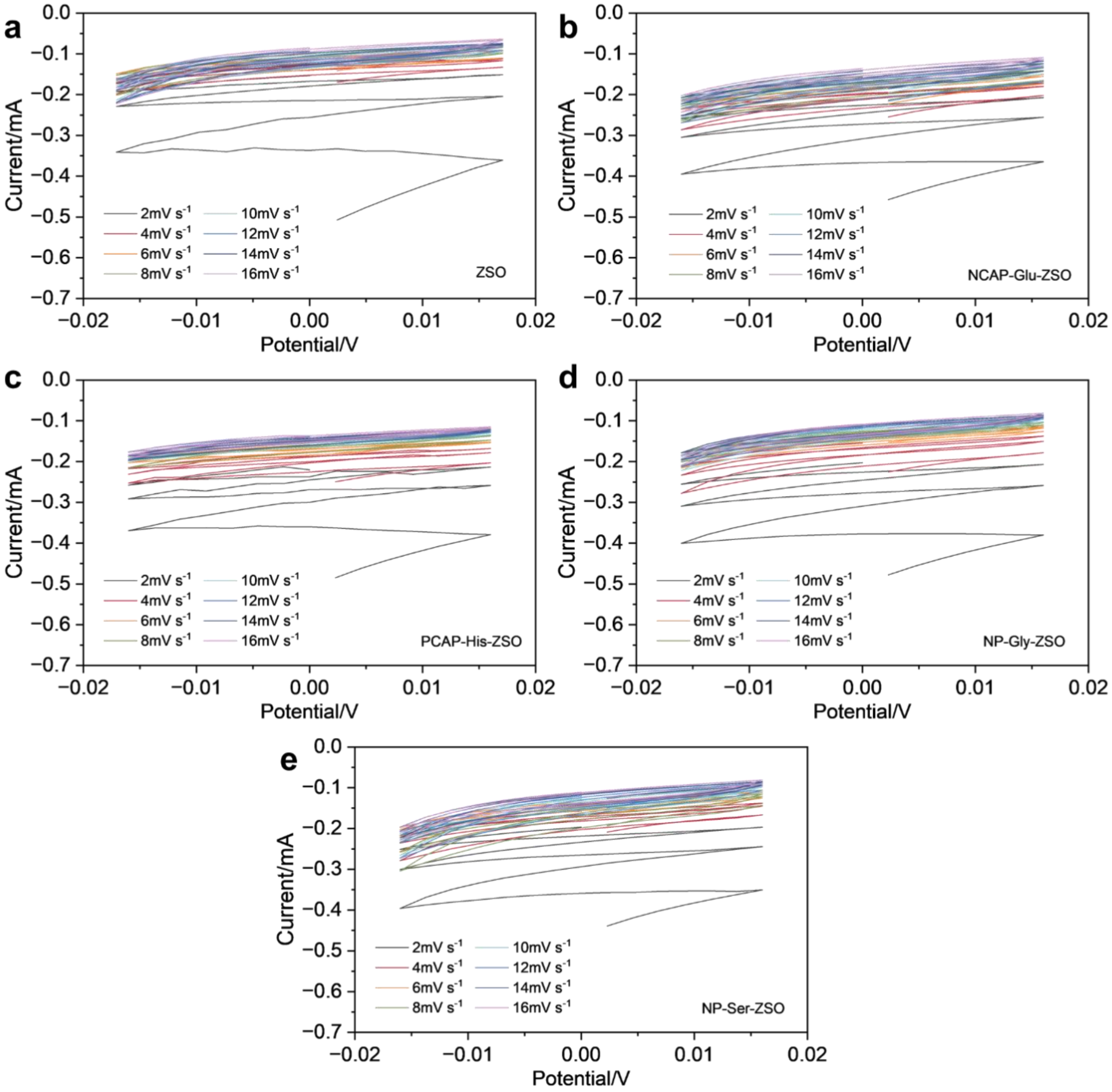

Fig. S4 Cyclic voltammetry (CV) curves using different scan rates within -15 to 15 mV in different electrolytes

The capacitance of EDL on an anode surface could be calculated by the defined equation:

$$C = \frac{i_c}{v}$$

Where i_c refers to the capacitance currents in CV scans and v refers to the scan rates of CV tests. Here, we chose $i_c=(i_{0v}+i_{1v})/2$, which means the half value of the current difference during the forward scan and negative scan at 0V. The scan rates were 2, 4, 6, 8, 10, 12, 14, 16, and 18 mV s^{-1}. Thus, the EDL capacitance on Zn anodes can be calculated as shown in Fig S4.

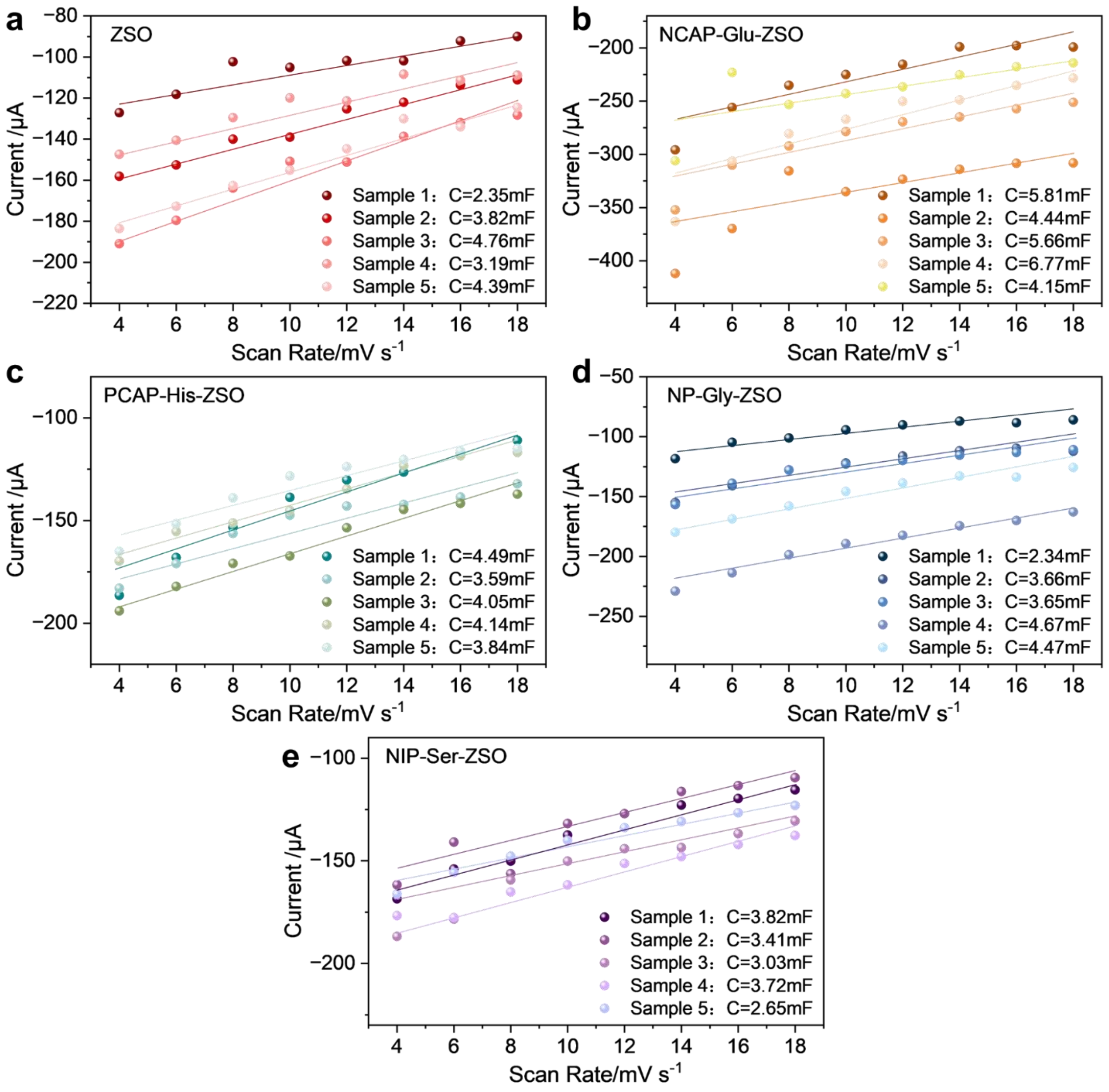

Fig. S5 EDL capacitance calculated in different electrolytes

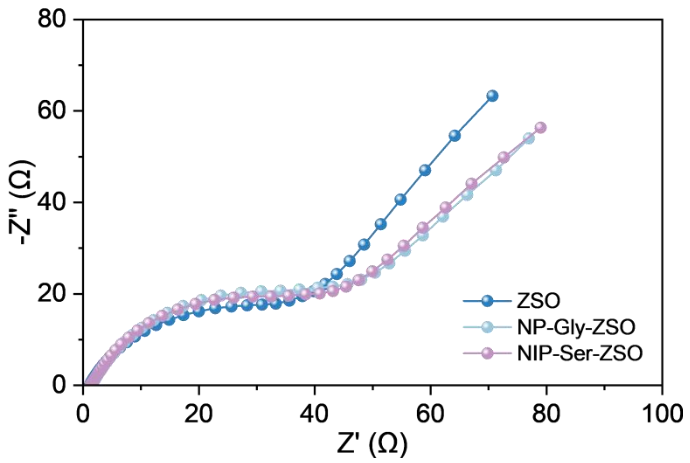

Fig. S6 Impedance diagrams of Zn|Zn symmetric cells using different electrolytes

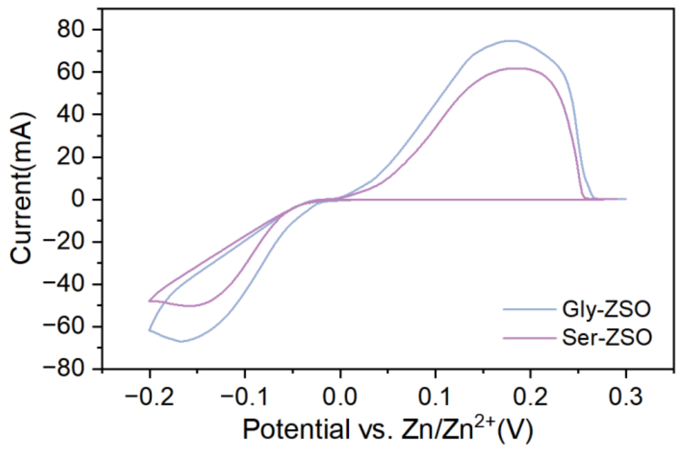

Fig. S7 Cyclic voltammetry curves of Zn|Cu asymmetric batteries in Gly-ZSO and Ser-ZSO electrolyte

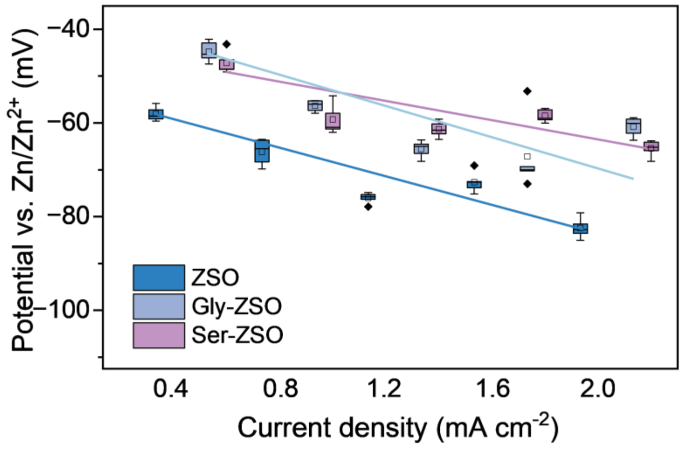

Fig. S8 Nucleation overpotential on Zn anode surface at different current densities in different electrolytes

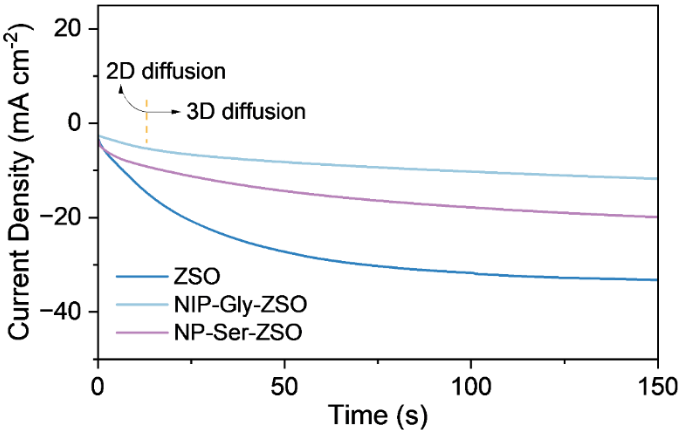

Fig. S9 Chronoamperograms of Zn metal in different electrolytes

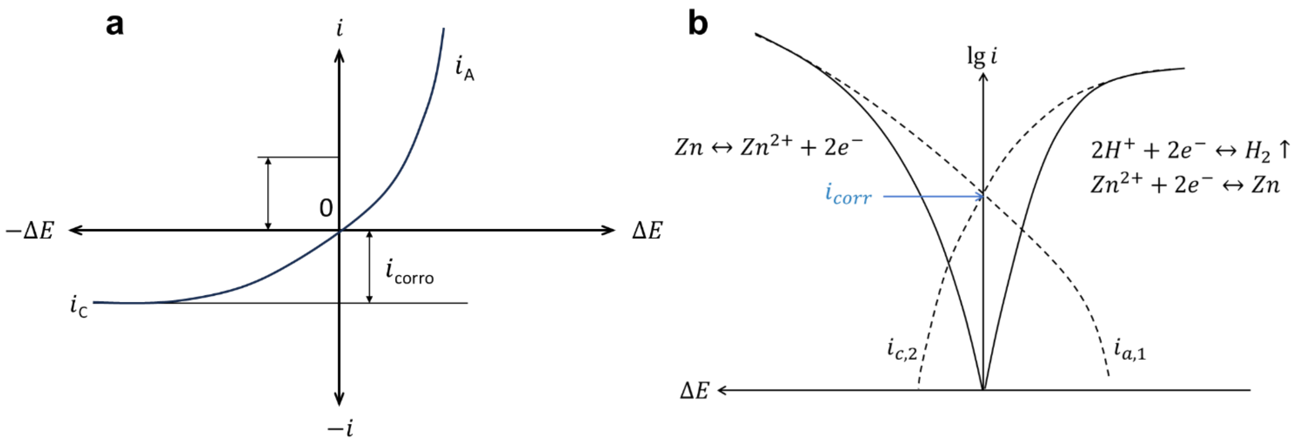

Fig. S10 a Polarization curve that the corrosion process is controlled by the cathodic diffusion process. **b** Corresponding polarization Tafel curve of Zn metal anode

To derive the formula of the relation between current density and potential on the anode, two assumptions are made:

(1) There exist only two electrode reactions on anode at the same time, which are the dissolution reaction and the depolarization reaction. The reaction rates of both two reactions are controlled by activation polarization, and the mass transfer process in the solution is very fast, thus the concentration polarization can be ignored;

(2) The corrosion potential is far from the equilibrium electrode potential of both the above two reactions to ensure the two reactions are under strong polarization, thus the inverse process of these reactions can be ignored.

Under the above assumed conditions, the kinetics of each electrode reaction can be expressed by the B-V formula, i.e.:

$$i_{a,1} = i_1^0 exp\frac{2.3(E-E_{e,1})}{b_{a,1}} \quad \text{(S1)}$$

$$i_{c,2} = i_2^0 exp[-\frac{2.3(E-E_{e,2})}{b_{c,2}}] \qquad (S2)$$

where, the subscript 1 represents the electrode reaction in the anode direction of metal anode dissolution, while the subscript 2 represents the electrode reaction in cathode direction of the depolarizer cathode reduction; $i_{a,1}$ is the dissolution rate of zinc; $i_{c,2}$ is the evolution rate of hydrogen; $b_{a,1} = \frac{2.3RT}{\beta_1 n_1 F}$; $b_{c,2} = \frac{2.3RT}{\alpha_2 n_2 F}$. When the corroded metal electrode is at the self-corrosion potential, the current measured externally would be zero, and the potential of corroded metal electrode is its corrosion potential E_{corro}, in which satisfies formula S3:

$$i_{a,1} = i_{c,2} = i_{corro} \qquad (S3)$$

$$i_{c,2} = i_1^0 exp\frac{2.3(E-E_{e,1})}{b_{a,1}} = i_2^0 exp\left[-\frac{2.3(E-E_{e,2})}{b_{c,2}}\right] = i_{corro} \qquad (S4)$$

That is, the absolute value of the current density of the anode reaction is equal to that of the cathode reaction on the metal electrode, while it is equal to the average corrosion current density of the metal electrode. The externally measured anodic polarization current density i_A and cathodic polarization current density i_C of the corroded metal electrode can be expressed as:

$$i_A = i_{a,1} - i_{c,2} \qquad (S5)$$

$$i_C = i_{c,2} - i_{a,1} \qquad (S6)$$

Formula S1 and S2 are substituted into formula S5 and S6, then:

$$i_A = i_1^0 exp\frac{2.3(E-E_{e,1})}{b_{a,1}} - i_2^0 exp[-\frac{2.3(E-E_{e,2})}{b_{c,2}}] \qquad (S7)$$

$$i_C = i_2^0 exp[-\frac{2.3(E-E_{e,2})}{b_{c,2}}] - i_1^0 exp\frac{2.3(E-E_{e,1})}{b_{a,1}} \qquad (S8)$$

Formula S4 is substituted into formula S7 and S8, then:

$$i_A = i_{corro}\{exp\frac{2.3(E-E_{e,1})}{b_{a,1}} - exp\left[-\frac{2.3(E-E_{e,2})}{b_{c,2}}\right]\} \qquad (S9)$$

$$i_C = i_{corro}\{exp[-\frac{2.3(E-E_{e,2})}{b_{c,2}}] - exp\frac{2.3(E-E_{e,1})}{b_{a,1}}\} \qquad (S10)$$

where, $E - E_{corro} = \Delta E$ is called the polarization value of corroded metal, and formulas S9 and S10 are the relationship of anode polarization curve and cathode polarization curve under electrochemical polarization, which is also called the basic formula of metal corrosion kinetics. If the positive and negative current signs are considered, the formulas S9 and S10 can be unified into the following formula:

$$i = i_{corro}\{exp\frac{2.3\Delta E}{b_a} - exp\left[-\frac{2.3\Delta E}{b_c}\right]\} \qquad (S11)$$

When the polarization $\Delta E = 0$, $i = 0$, the corrosion system is at the open state; when the polarization $\Delta E > 0$, $i > 0$, the corroded metal electrode is at the state of anodic polarization; when the polarization $\Delta E < 0$, $i < 0$, the corroded metal electrode is at the state of cathodic polarization. If the cathodic reaction rate of the corrosion process is influenced by the diffusion process of the depolarizer in the electrolyte, the formula needs to be further supplemented. When there exists concentration polarization, the relationship between the absolute value of the current density and the electrode potential is as follows:

$$|i_C| = (1 - \frac{|i_C|}{i_L}) i_C^0 exp[-\frac{2.3(E - E_{c,e})}{b_c}] \quad \text{(S12)}$$

where i_L is the limiting diffusion current density. Substituting the relationship of $|i_C| = i_{corro}$ when $E = E_{corro}$ into formula S12, then:

$$|i_C| = \frac{i_{corro}\exp(-\frac{2.3\Delta E}{b_c})}{1 - \frac{i_{corro}}{i_L}[1 - \exp(-\frac{2.3\Delta E}{b_c})]} \quad \text{(S13)}$$

Thus, the polarization curve formula of the corroded metal electrode is obtained:

$$i = i_{corro}\{exp\frac{2.3\Delta E}{b_a} - \frac{\exp(-\frac{2.3\Delta E}{b_c})}{1 - \frac{i_{corro}}{i_L}[1 - \exp(-\frac{2.3\Delta E}{b_c})]}\} \quad \text{(S14)}$$

Under certain conditions, when the rate of the corrosion process is controlled by the cathodic diffusion process (Fig S10), the corrosion current density is equal to the absolute value of the diffusion current density of the cathodic reaction, which means $i_{corro} \approx i_L$. In this case, it can be obtained from formula S14:

$$i = i_{corro}(exp\frac{2.3\Delta E}{b_a} - 1) \quad \text{(S15)}$$

Therefore, formula S15 represents the polarization curve controlled by concentration polarization, and the corresponding curve is shown in Fig S10.

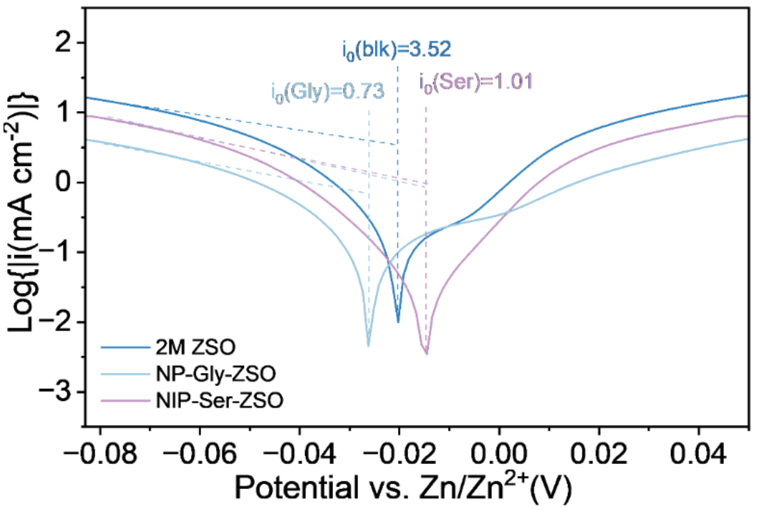

Fig. S11 Tafel curves of Zn|Zn symmetric cells in different electrolytes

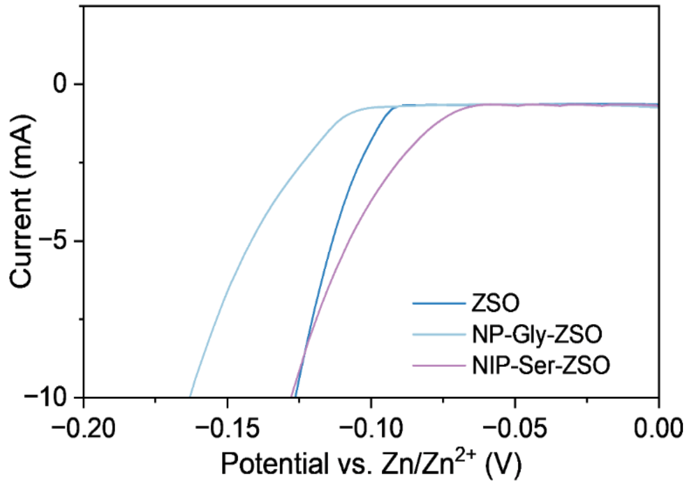

Fig. S12 Linear sweep voltammetry curves of Zn anode in different electrolytes

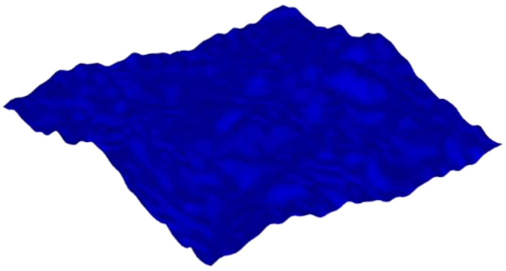

Fig. S13 The initial state of Zn anode surface in FEM simulation of the random 3D surface with roughness of 18.85 μm and size of 100 μm×100 μm

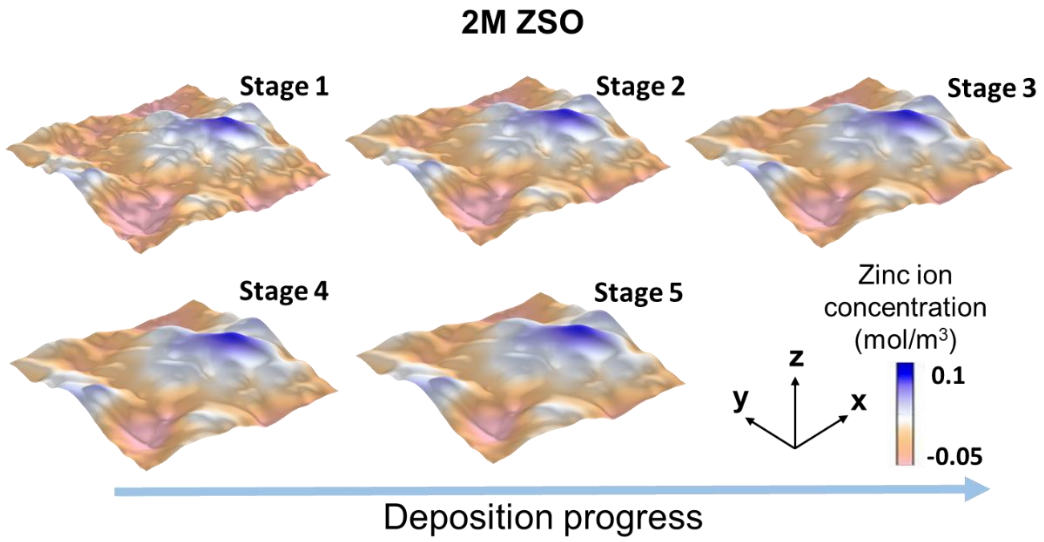

Fig. S14 FEM simulation of Zn^{2+} concentration distribution changing progress on Zn anode surface during deposition at 1 mA cm^{-2} with a capacity of 1 mAh cm^{-2} in ZSO electrolyte

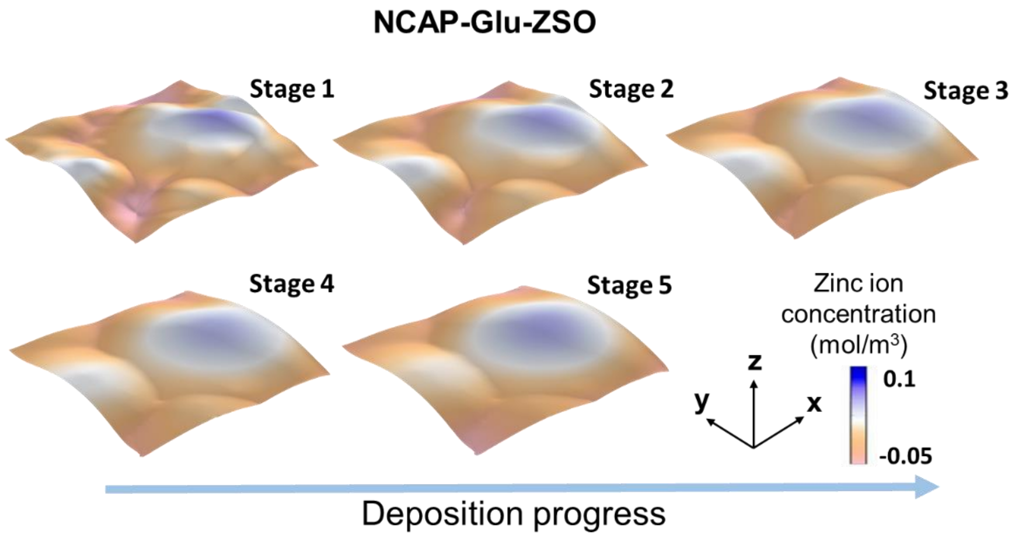

Fig. S15 FEM simulation of Zn^{2+} concentration distribution changing progress on Zn anode surface during deposition at 1 mA cm^{-2} with a capacity of 1 mAh cm^{-2} in NCAP-Glu-ZSO electrolyte

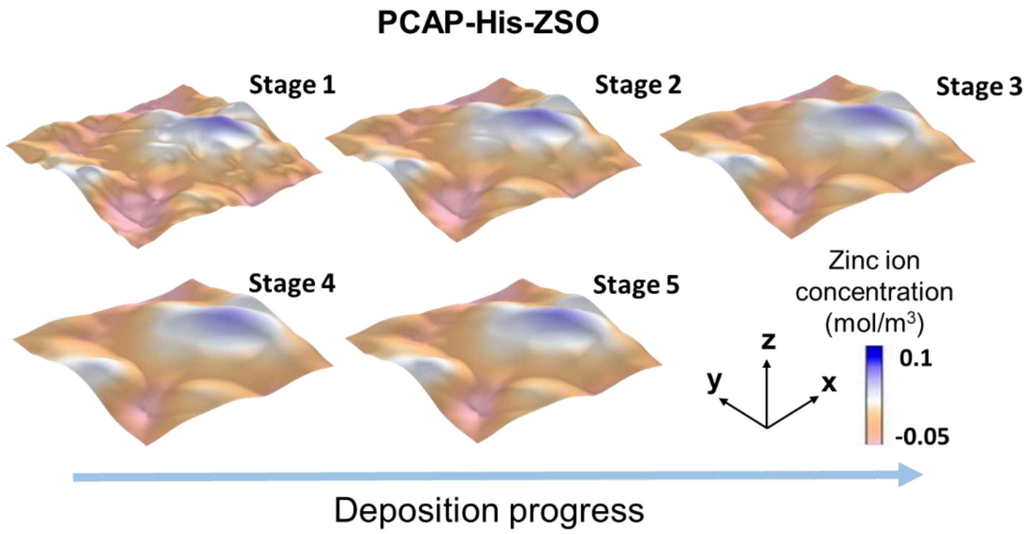

Fig. S16 FEM simulation of Zn^{2+} concentration distribution changing progress on Zn anode surface during deposition at 1 mA cm^{-2} with a capacity of 1 mAh cm^{-2} in PCAP-His-ZSO electrolyte

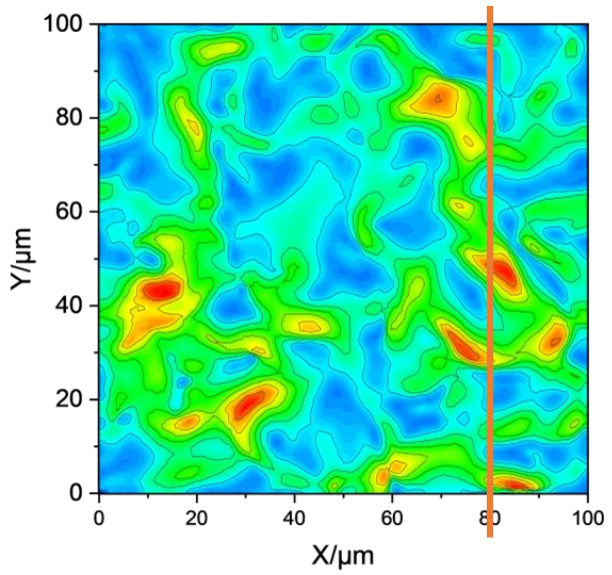

Fig. S17 Diagram of the selection of longitudinal current density distribution on Zn anode surface of Fig. 3g: x=80 μm, width=0.1 μm

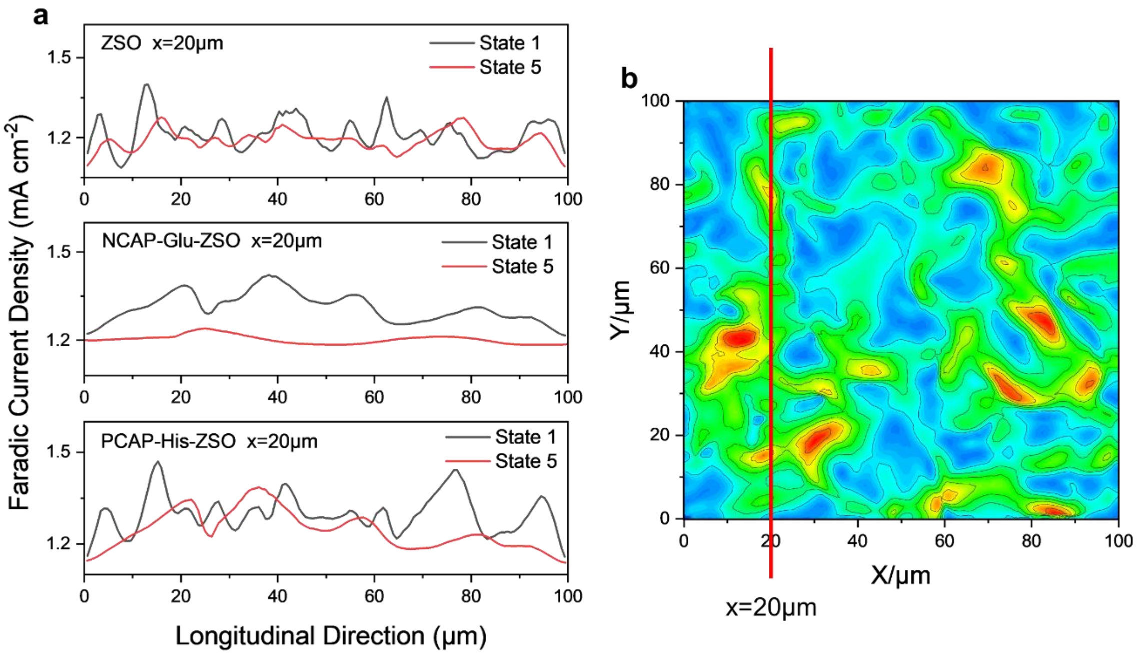

Fig. S18 a Longitudinal current density distribution of Zn anode surface in different electrolytes **b** at the location of x=20 μm, width=0.1 μm

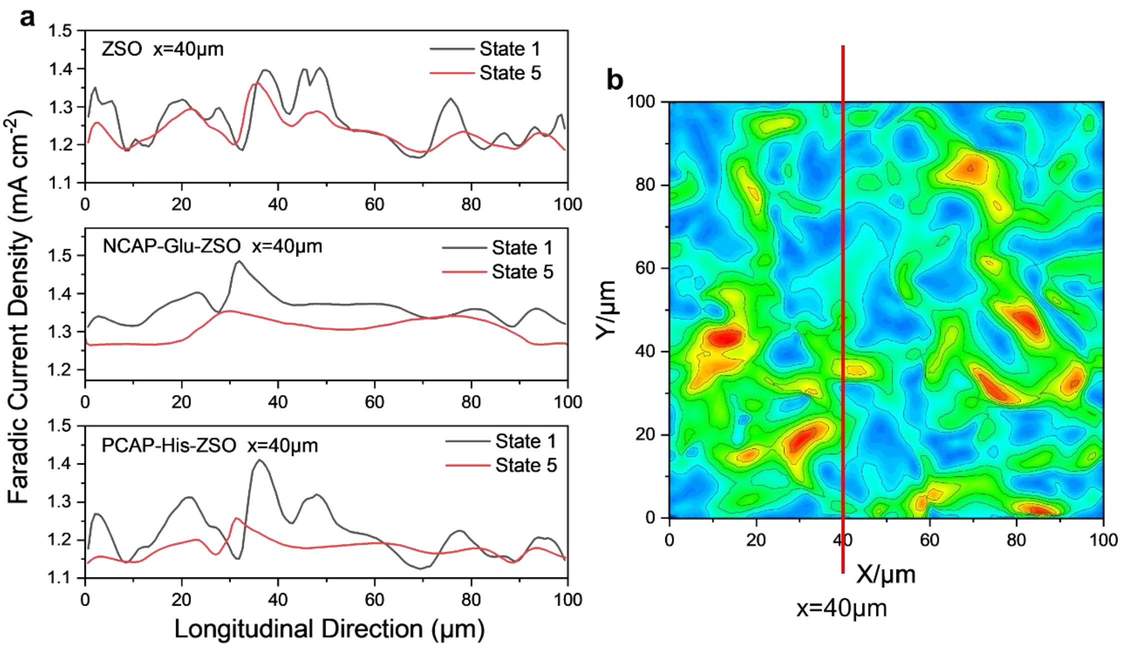

Fig. S19 a Longitudinal current density distribution of Zn anode surface in different electrolytes **b** at the location of x=40 μm, width=0.1 μm

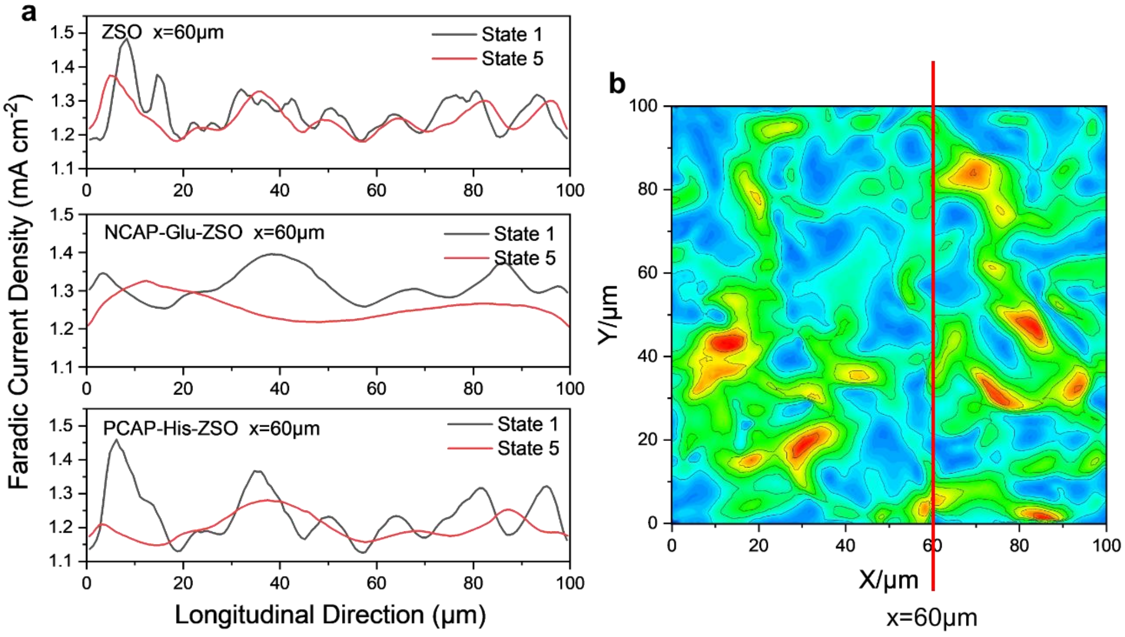

Fig. S20 a Longitudinal current density distribution of Zn anode surface in different electrolytes **b** at the location of x=60 μm, width=0.1 μm

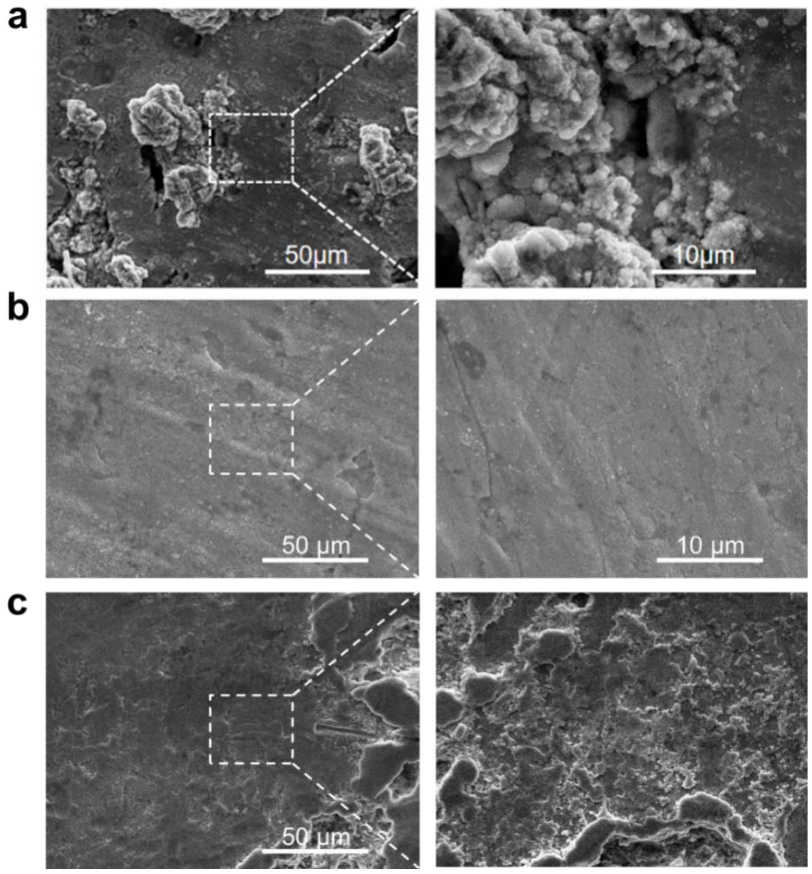

Fig. S21 SEM images of Zn metal anode after 10 cycles in **a** 2M ZSO; **b** 0.1M Glu-ZSO; **c** 0.1M His-ZSO at 1 mA cm^{-2} with a capacity of 1mAh cm^{-2}

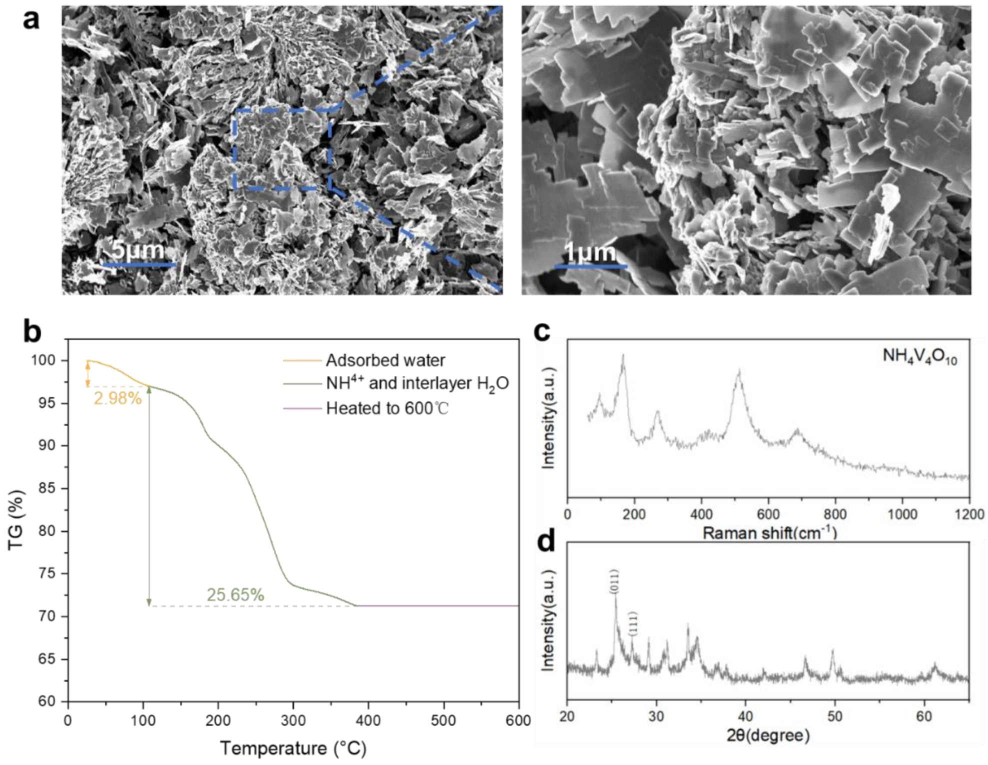

Fig. S22 Characterization of NVO powder: **a** SEM images; **b** Thermogravimetric analysis (TGA); **c** Raman Spectra; **d** XRD spectra

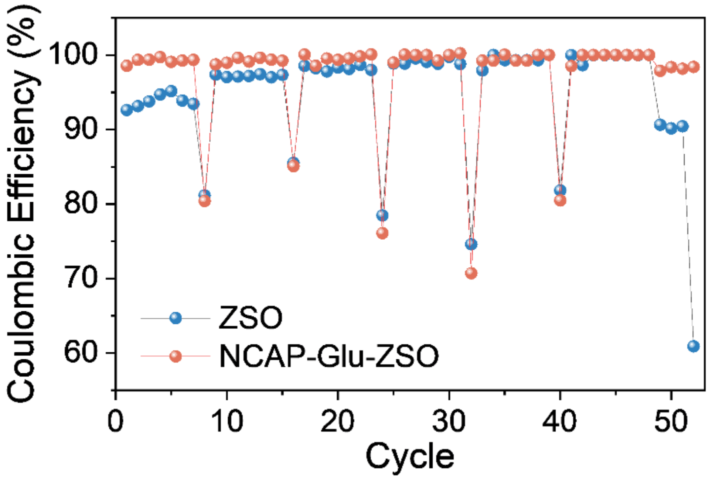

Fig. S23 Coulombic efficiency of $NH_4V_4O_{10}$|Zn cells at various current densities from 0.1 to 3 A g^{-1}

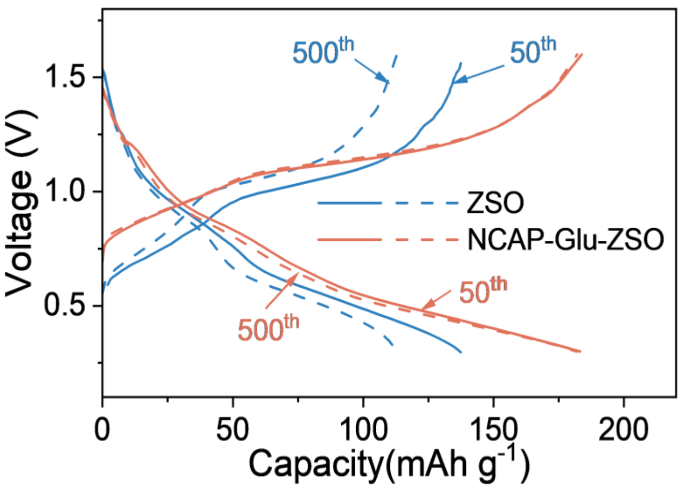

Fig. S24 Capacity-voltage curves of different cycles of $NH_4V_4O_{10}$|Zn cells with/without glutamate at a current density of 2 A g^{-1}
